# Progress on AlGaN-based solar-blind ultraviolet photodetectors and focal plane arrays

**DOI:** 10.1038/s41377-021-00527-4

**Published:** 2021-04-30

**Authors:** Qing Cai, Haifan You, Hui Guo, Jin Wang, Bin Liu, Zili Xie, Dunjun Chen, Hai Lu, Youdou Zheng, Rong Zhang

**Affiliations:** 1 Key Laboratory of Advanced Photonic and Electronic Materials, School of Electronic Science and Engineering, Nanjing University, Nanjing, 210093 China; 2 Collaborative Innovation Center for Optoelectronic Semiconductors and Efficient Devices, Department of Physics, Xiamen University, Xiamen, 361005 China; 3 Institute of Future Display Technology, Tan Kah Kee Innovation Laboratory, Xiamen, 361102 China

**Keywords:** Optics and photonics, Optical materials and structures

## Abstract

Solar-blind ultraviolet (UV) photodetectors (PDs) have attracted tremendous attention in the environmental, industrial, military, and biological fields. As a representative III-nitride material, AlGaN alloys have broad development prospects in the field of solar-blind detection due to their superior properties, such as tunable wide bandgaps for intrinsic UV detection. In recent decades, a variety of AlGaN-based PDs have been developed to achieve high-precision solar-blind UV detection. As integrated optoelectronic technology advances, AlGaN-based focal plane arrays (FPAs) are manufactured and exhibit outstanding solar-blind imaging capability. Considering the rapid development of AlGaN detection techniques, this paper comprehensively reviews the progress on AlGaN-based solar-blind UV PDs and FPAs. First, the basic physical properties of AlGaN are presented. The epitaxy and p-type doping problems of AlGaN alloys are then discussed. Diverse PDs, including photoconductors and Schottky, metal–semiconductor–metal (MSM), p-i-n, and avalanche photodiodes (APDs), are demonstrated, and the physical mechanisms are analyzed to improve device performance. Additionally, this paper summarizes imaging technologies used with AlGaN FPAs in recent years. Benefiting from the development of AlGaN materials and optoelectronic devices, solar-blind UV detection technology is greeted with significant revolutions.

## Abstract (web summary)

Summarizing recent advances in the processing and properties of AlGaN-based solar-blind UV photodetectors and focal plane arrays as well as AlGaN growth and doping techniques.

## Graphical abstract

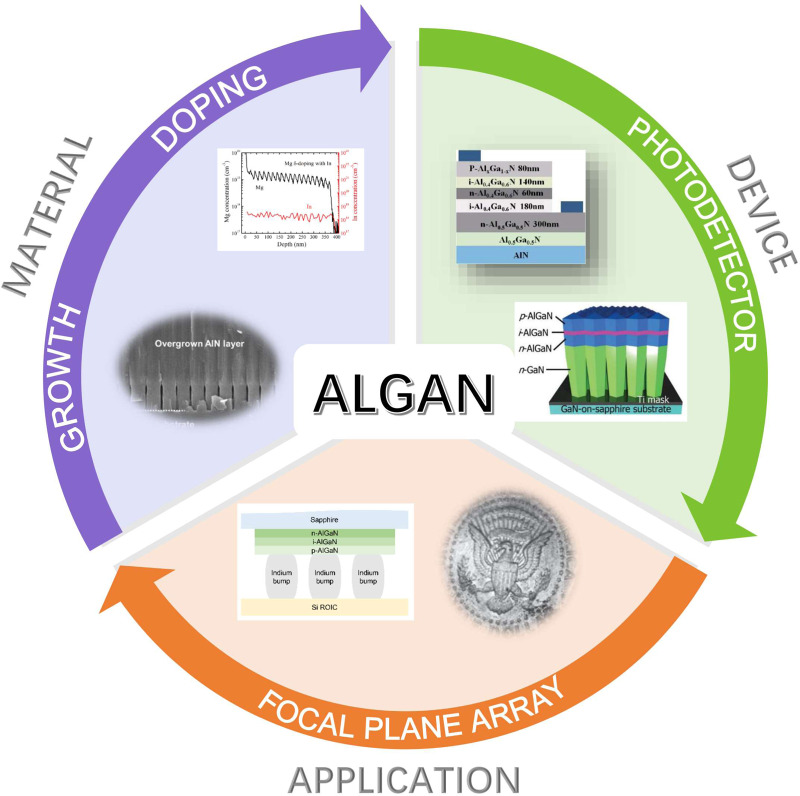

## Introduction

Ultraviolet (UV) radiation, which covers the electromagnetic spectrum from 400 to 10 nm, can be divided into four subdivisions: UVA (320–400 nm), UVB (280–320 nm), UVC (200–280 nm), and VUV (vacuum UV, 10–200 nm). The sun is the primary source of UV light, and UVC light it typically absorbed by the ozonosphere when it passes through the atmosphere. Thus, no UVC photons exist naturally within the Earth’s atmosphere. Therefore, the UVC solar spectrum is also called the solar-blind UV waveband^1–3^. This feature ensures that the detection of solar-blind UV photon signals within the Earth’s atmosphere is not affected by background radiation from sunlight, which gives the solar-blind UV detecting potential applications in early missile threat warning and tracking, environmental monitoring, engine monitoring, flame detection and monitoring, non-line-of-sight communications, etc.^4–12^.

Group-III nitride semiconductors exhibit superior properties, such as a wide energy bandgap, large thermal conductivity, high carrier mobility, small dielectric constant, strong anti-radiation ability, and good chemical stability^13–17^. Due to these superior properties, III-nitride semiconductors can be applied in extreme environments^18^, solid-state lighting and displays^19^, short-wavelength lasers^20–22^, and optical detection^23–26^. III-nitride semiconductors primarily include GaN, AlN, and InN along with their ternary and quaternary alloys AlGaN, InGaN, and AlGaInN^27–32^. Among these materials, the AlGaN ternary alloy semiconductor can tune its bandgap in the range of 3.4–6.2 eV by changing the Al component, covering the UVA, UVB, and UVC wavelength bands of 200–365 nm. Additionally, AlGaN exhibits a high specific detectivity approximately 10^12^ cm Hz^1/2^ W^−1 33^. Based on these outstanding optoelectronic characteristics, AlGaN ternary alloys exhibit marked advantages in promoting the evolution of UV photodetectors (PDs), particularly in fabricating intrinsic solar-blind UV detectors^34,35^.

In 1998, O Ambacher concluded various results of Group-III nitride semiconductor devices^36^. Due to the rapid development of blue light-emitting diodes (LEDs), III-nitride based blue-emitting lasers, high power transistors, and PDs have also gradually attracted more attention. In 2001, E Munoz et al. reviewed the basic structure of a III-nitride UV photodetector^37^ and focused on the essential physical mechanisms of devices with different structures and preliminarily emphasized that UV imaging will drastically change various aspects of civilian and military fields. In 2005, Khan et al. provided a review of advances in III-nitride LEDs and laser diodes (LDs)^38^. The development of AlGaN LEDs also promotes progress in the epitaxial techniques of subsequent AlGaN-based photodetect (PD) materials. In 2011, Razeghi summarized the developments of III-nitride materials in critical spectral regimes from UV to terahertz wavelengths^39^. Among these materials, AlGaN alloys play a significant role in the LEDs, avalanche photodiodes (APDs), single photon detectors, and focal plane arrays (FPAs). In 2015, Alaie et al. described the advantages of different UV PDs such as AlGaN, ZnO, MgZnO, SiC, diamond, and other material compound PDs^40^, and also analyzed the state of UV detection from the aspect of material. In 2018, Li. et al. reviewed AlGaN-based materials and UV devices in detail^41^. Various devices and application scenarios of AlGaN are reviewed in their work. Despite considerable effort, more robust content in AlGaN solar-blind UV detection are required for better understanding the development of this field and finding out the problems needed to be solved in the future.

In terms of FPAs, Dupuis et al. concluded their work on GaN-based UV APDs and FPA imaging applications^42^. However, their fabricated devices were primarily composed of GaN and low-Al-content AlGaN, and their detection and imaging wavelengths were primarily located in the UVA band. High-Al-content AlGaN material fabrication has always been a serious problem. In addition, although different research groups have reported corresponding UV FPAs in conjunction with their UV detectors, recent summaries of AlGaN solar-blind detection and FPAs are inadequate.

In recent years, AlGaN-based materials and their UV PDs have been rapidly developed^43–46^. The fabrication techniques of common Schottky and p-i-n structure AlGaN UVA PDs are relatively mature^47–52^. However, AlGaN-based solar-blind UV PDs and FPAs remain to be improved due to uniformity problems in materials and devices^53–62^. To perform solar-blind UV detection, the bandgap of AlxGa1-xN must exceed 4.42 eV (Al composition: ~0.4). Due to the low surface mobility of Al adatoms and large lattice mismatch between AlN and GaN^63–71^, the epitaxy of high-Al-content AlGaN alloys with high crystal quality is one of the primary problems in the development of AlGaN-based solar-blind detectors^72–74^. Another problem is the low p-type doping efficiency of AlGaN^75–81^, and it is difficult to obtain high-hole-concentration and high-conductivity p-AlGaN materials. This phenomenon can be attributed to the high activation energy of the Mg acceptor in the AlGaN alloy^82^. In addition to material issues, device design and key FPA techniques are also the primary factors that must be considered in the development of AlGaN-based solar-blind detection technology.

**Fig. 1:**
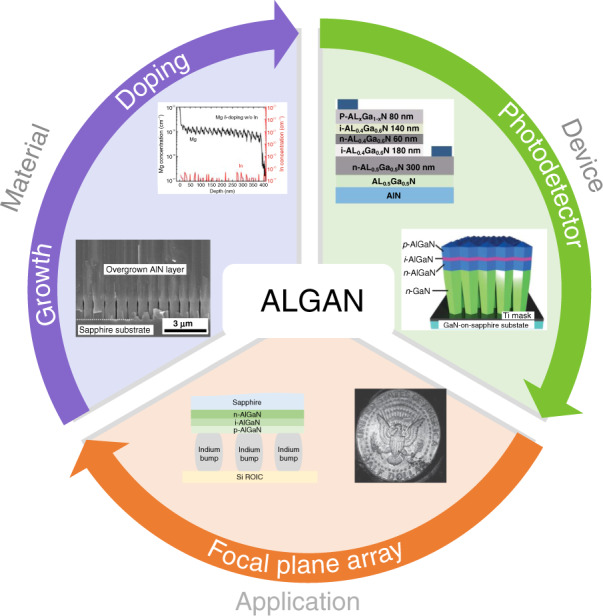
Diagram of this review. Including the material section (AlGaN growth and doping), device section (AlGaN solar-blind photodetector), and application section (AlGaN focal plane array)

This review summarizes recent advances in the processing and properties of AlGaN-based solar-blind UV PDs and FPAs. In the following sections, epitaxial growth and doping techniques of AlGaN are presented. Then, the third section primarily focuses on the development of AlGaN solar-blind UV PDs, including the progress of various detectors, performance improvement methods, and internal physical mechanisms. Moreover, the advances in AlGaN-based solar-blind FPAs and imaging techniques are reviewed to better understand solar-blind technology for versatile applications (Fig. 1).

## Epitaxial growth and doping of AlGaN

The high crystalline quality of AlGaN is the basis of high-performance AlGaN-based devices. The growth methods of III–V compound semiconductors can be primarily divided into two categories—the monoblock single crystal growth method, and the epitaxial growth method, which groups materials with the same crystalline orientation on the substrate. In monoblock method, the Bridgman method involves placing the material in a vertically crucible with a tip and then solidifying it directionally from the tip of the crucible through a sharply gradient temperature. Directional solidification of the material can be achieved by a moving furnace or a moving crucible. The horizontal Bridgman method was also developed for GaAs growth.

With regard to III–V compound semiconductors with volatile components, the V components tend to overflow from the melt due to dissociation pressure at their melting point. To prevent this phenomenon and allow the semiconductor compound material to be grown as a single crystal in a conventional czochralski (CZ) furnace, the surface of the compound semiconductor solution can be covered with a transparent layer with high chemical stability, a low melting point, and a high boiling point (e.g., B_2_O_3_). Concurrently, the single crystal furnace is filled with inert gas. The compound semiconductor can then be grown as a single crystal by the liquid encapsulation czochralski (LEC) process, as silicon and germanium are grown by the CZ method.

However, these methods typically cannot avoid contamination by impurities from quartz crucibles and heating systems. Due to the high melting point of GaN/AlGaN material (>1700 °C), it is also difficult to prepare AlGaN bulk single crystal materials using these methods. Even with high-temperature (HT) and high-pressure technologies, only powdered and needle-like AlGaN crystals can be prepared. The growth of thin film AlGaN materials still depends on the development of epitaxial technology.

There are three types of epitaxy methods: liquid phase epitaxy (LPE), molecular beam epitaxy (MBE), and vapor phase epitaxy (VPE). The LPE method is easy to operate, and a single crystal grows at low temperature and fast speed. However, the LPE method can only fabricate a thin epitaxy layer with limited thickness. It is also difficult to purposefully change the gradient of impurity concentrations and the type of conductivity during the growth process intensively. Hydride vapor phase epitaxy (HVPE) can grow III-nitrides rapidly. However, it is difficult to accurately control the film thickness with this method, and the reaction gas is corrosive to the equipment, which effects the purity of GaN(AlGaN) materials. MBE can precisely control the thickness of the growth film. Additionally, the growth temperature is lower than that of the VPE and LPE methods. Doping and composition of material can be modulated in this way. However, the low growth rate, complex equipment, and high cost impede the application of the MBE method in large-scale industrial production. Metal-organic vapor phase epitaxy (MOVPE) achieves a moderate growth rate and can accurately control the film thickness, which is particularly suitable for large-scale industrial production. MOVPE is also the most widely used method to growth of GaN(AlGaN) materials and high-performance devices.

However, the epitaxy of AlGaN material poses several serious problems. AlGaN material is typically grown on an AlN/sapphire substrate^83–87^. Due to the large lattice mismatch of 13.2% between AlN and sapphire, the AlN template presents many dislocations, which affect the epitaxial AlGaN crystal quality^88–91^. Meanwhile, AlGaN epitaxy requires temperatures in excess of 1300 °C. The large thermal expansion mismatch in the AlGaN/AlN/sapphire structure results in heat-induced stress, which is released by dislocations^89,91–93^. Additionally, because the surface adhesion coefficient of Al atoms is much higher than that of Ga atoms, dense islands tend to form during nucleation growth. To minimize the free energy of the system, two adjacent islands are combined, thus leading to tensile stress^94^. Because tensile stress is inversely proportional to the island size, the AlGaN film tends to crack due to excessive tensile stress, deteriorating the quality of the film. Also, the Al source provider trimethyl aluminum (TMA) is relatively active and reacts with ammonia gas above the substrate to form large numbers of microparticles before reaching the growth surface. The microparticles generated by the pre-reaction fall on the surface of material and produce many defects.

The realization of effective doping is also a basic requirement of semiconductor device fabrication^76,95–99^. For (Al)GaN, p-type doping of nitride is an important factor that restricts the development of GaN-based devices. Although breakthroughs have been made in p-GaN^100–102^, there are difficulties in p-type doping of high-Al-content AlGaN because the activation energy of the Mg acceptor increases with increasing Al content. Therefore, it is important to study the doping characteristics of nitride semiconductor materials to improve the performance of AlGaN-based devices.

### Epitaxy of high-Al-content AlGaN

To improve the crystalline quality of high-Al-content AlGaN epitaxial thin films, researchers have tried various methods to control stress to reduce the dislocations during the material growth process. Mudu et al.^103^ incorporated a low temperature (840 °C) AlN nucleation layer onto the c-plane sapphire substrate. Subsequently, an AlN buffer layer grown at high temperature (1150 °C) is employed to convert the island-like three-dimensional growth mode at low temperatures into a two-dimensional growth mode. Several dislocations are then bent to form a closed loop and disappear. Concurrently, the AlN buffer layer can adjust the stress and alleviate the subsequent stress accumulation of the AlGaN epitaxial film, which is conducive to obtaining better high-quality, crack free, and high-Al-content AlGaN material. Adivarahan et al.^97^ proposed that a small amount of In elements can be added as a lubricant on the growth surface to enhance the migration of Al atoms, thus alleviating the defects resulting from strong adhesion coefficient of Al adatoms. Codoping of In elements can also significantly increase the effective doping concentration of n-AlGaN. Using this method, they obtained a smooth, low-roughness surface morphology, and significantly reduced the screw dislocation and edge dislocation density of the AlGaN material.

**Fig. 2:**
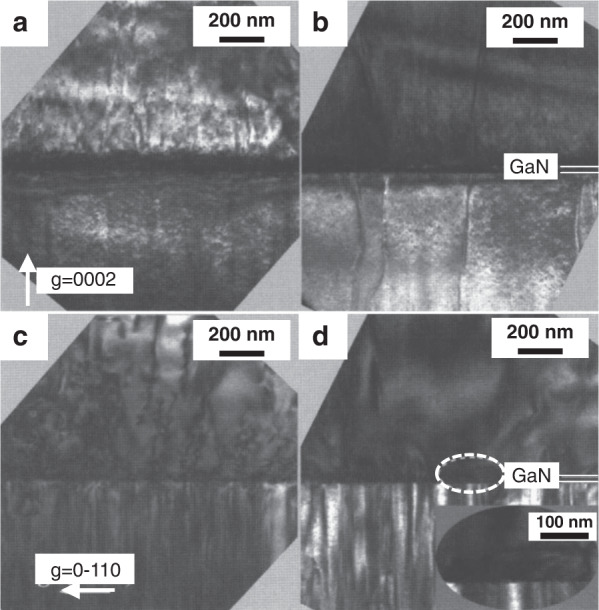
Cross-sectional TEM images for the AlGaN layers grown on AlN/sapphire templates. Samples without (**a**, **c**) and with (**b**, **d**) a 25 nm HT-GaN interlayer. **a**, **b** are measured with diffraction vector *g* = (0002) to image screw-component threading dislocations. **c**, **d** with *g* = (0–110) to image edge-component threading dislocations. The inset of Fig. 2**d** is an enlarged image of the dislocation, corresponding to the dashed circle. Reprinted with permission from Jiang et al.^104^. Copyright 2005 American Institute of Physics

Interlayer modulation can effectively grow AlGaN materials. Jiang et al.^104^ inserted a 25-nm-thick HT GaN layer between the AlN template and the AlGaN layer to block dislocations from penetrating through the AlGaN layer. They found that the HT-GaN layer can effectively block the vertical edge dislocations (Fig. 2c, d), while the screw dislocation density remains unchanged (Fig. 2a, b). This method can reduce the total dislocation density of the AlGaN material by an order of magnitude.

**Fig. 3:**
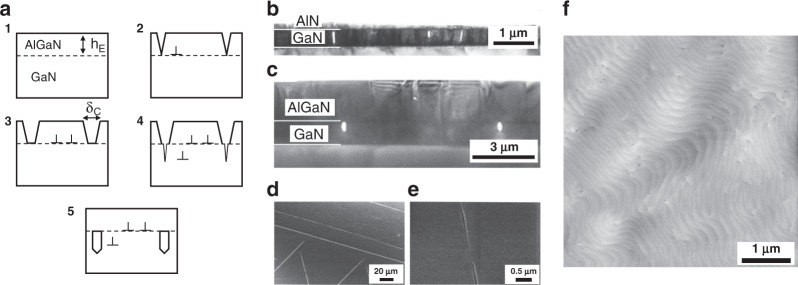
Control of stress and strain during AlGaN/AlN epitaxial growth. **a** Schematic diagram of relaxation process: (1) coherent growth below the critical thickness; (2) AlGaN cracks and MDs are introduced at AlGaN/GaN interface; (3) Relaxation resulted from dislocations enlarges the crack aperture; (4) cracks propagate to the GaN layer, MDs relaxing in GaN; (5) lateral growth buries cracks. Cross-sectional XTEM images of **b** MBE-grown AlN and **c** MOCVD-grown Al_0.2_Ga_0.8_N films on GaN. **d** SEM images of a 500-nm-thick cracked MOCVD-grown Al_0.2_Ga_0.8_N film on GaN. **e** Enlarged SEM image of crack overgrowth. **f** AFM image of a crack free Al_0.2_Ga_0.8_N film. Reprinted with permission from Bethoux et al.^105^. Copyright 2003 American Institute of Physics

The control of stress and strain during epitaxial growth is also one of the primary factors that must be considered. Bethoux et al.^105^ prepared a thick fully plastic strain relaxed AlGaN cracking layer on the GaN template, and then buried cracks via lateral epitaxial growth to achieve a high-quality AlGaN film with a smooth surface and no cracks. Figure 3a shows the strain-relaxation process. The cracks of the AlGaN film introduce misfit dislocations (MDs) at the AlGaN/GaN interface. A significant opening of cracks is formed due to the contraction of AlGaN stripes. The tensile stress leads to crack propagation into the inserted GaN layer. Crack edges in the GaN layer allow the nucleation of MDs and AlGaN lateral growth over cracks can bury MDs. Finally, a smooth and crack free film with high crystalline quality is obtained. This method is also suitable for AlN epitaxy. Figure 3b, c confirm the burying of cracks by regrowth. Voids can be distinctly observed in the inserted GaN layer, as shown in Fig. 3c. Figure 3d illustrates the cracked MOCVD-grown Al_0.2_Ga_0.8_N film on GaN. The enlarged SEM image also verifies crack overgrowth, as shown in Fig. 3e. An AFM image of the smooth Al_0.2_Ga_0.8_N film exhibits a marked step-flow morphology. The density of threading dislocations is estimated to be 5 × 10^8^ cm^−2^. The root-mean-square (RMS) roughness of the film is below 1 nm in a 15 × 15 μm^2^ scan.

**Fig. 4:**
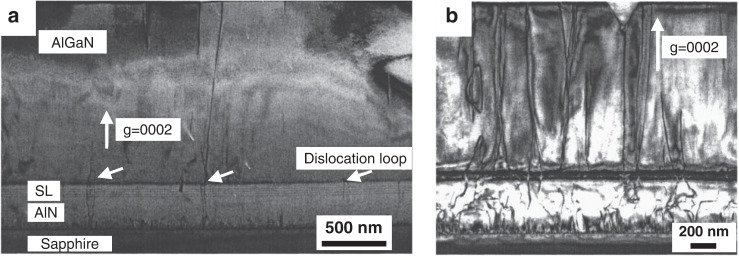
AlGaN/AlN superlattice employed on the AlGaN growth. Cross-section TEM images [vector: *g* = (0002)] showing screw-component TDs in n-Al_0.55_Ga_0.45_N with (**a**) and without (**b**) superlattice insertion. Reprinted with permission from Sun et al.^108^. Copyright 2005 American Institute of Physics

Zhang et al.^106,107^ inserted an AlGaN/AlN superlattice (SL) layer to adjust the stress of the AlGaN film during the epitaxial growth of AlGaN. This growth technique can be used to grow thick crack-free AlGaN films. Sun et al.^108^ also used pulsed atomic layer epitaxy (PALE) to grow short-period SL structures. The PALE method can enhance the migration of Al adatoms and reduce parasitic pre-actions. The TEM micrographs in Fig. 4 show screw-type threading dislocations for two 1-μm-thick n^+^-Al_0.55_Ga_0.45_N layers. Samples are grown on AlN buffer with and without the SL structure. Figure 4a shows that there are few threading dislocations at the interfaces between AlN, SL, and top n-AlGaN. Instead, dislocation loops in Fig. 4a at the interface between AlGaN and SL are formed to eliminate threading dislocations after inserting the SL structure. Conversely, Fig. 4b shows large amounts of dislocations in the n^+^-Al_0.55_Ga_0.45_N layer without SL. The overall screw-type threading dislocations are estimated to be below 3 × 10^8^ cm^−2^. Compared to direct epitaxy (dislocation density: ~5 × 10^9^ cm^−2^), the number of dislocations is reduced by more than an order of magnitude.

**Fig. 5:**
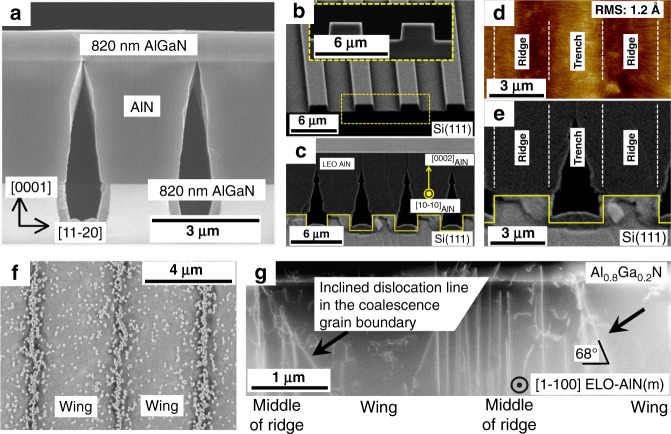
Epitaxial lateral overgrowth for AlGaN fabrication. **a** Cross-sectional secondary electron image of AlGaN grown on an AlN ELO template. Reprinted with permission from Kueller et al.^117^. Copyright 2010 Elsevier B.V. **b**, **c** SEM images of the stripe-patterned AlN/Si(111) template. **d** AFM image of the ELO-AlN layer grown on the patterned Si template and corresponding cross-sectional SEM image of the patterned structure (**e**). Atomic steps are observed over the trenches. Reprinted with permission from Cicek et al.^118^. Copyright 2013 American Institute of Physics. **f** Plan-view SEM image of n-Al_0.8_Ga_0.2_N grown on ELO-AlN. **g** Cross-sectional annular dark-field STEM image presents that the defect distribution in the ELO-AlN-AlGaN structure. Reprinted with permission from Mogilatenko et al.^116^. Copyright 2014 Elsevier B.V

Epitaxial lateral overgrowth (ELO) is also an important method for growing high-Al-content AlGaN and AlN materials^109–113^. The commonly used method is to grow AlGaN on the pattern template^114–116^. Typically, as the epitaxial AlGaN film thickness increases, the dislocation density decreases. Kueller et al.^117^ proposed epitaxial overgrowth of AlGaN on a structured AlN/sapphire template, as shown in Fig. 5a. They also found that stripes parallel to the [1–100] direction are a more promising pattern for AlGaN coalescence in their growth conditions. Cicek et al.^118^ reported an AlGaN-based UV detector grown on a patterned Si(111) template (Fig. 5b) and used PALE technique to facilitate the epitaxial lateral growth of AlN, as shown in Fig. 5c. Due to the slow epitaxial lateral growth rate and high aspect ratio trench, air voids form at the trench location after the AlN has grown 8.5 μm vertically. The air voids mitigate crackings and reduce the generation of dislocations in the AlN template. Figure 5d shows an AFM image of a coalesced AlN surface in a 5 × 10 μm scan. Step-flow morphology is shown with an RMS roughness of 0.12 nm in the trench regions. However, the ridge regions exhibit different characteristics. Based on this phenomenon, Mogilatenko et al.^116^ studied the dislocation evolution and distribution in thick AlN layers with the ELO method. As shown in Fig. 5g, the threading dislocation inclination contributes to the compensation of compressive strain in the growth process of Al_0.8_Ga_0.2_N on the ELO-AlN template. Therefore, the wing (trench) regions exhibit lower defect densities than the ridge regions (Fig. 5f).

**Fig. 6:**
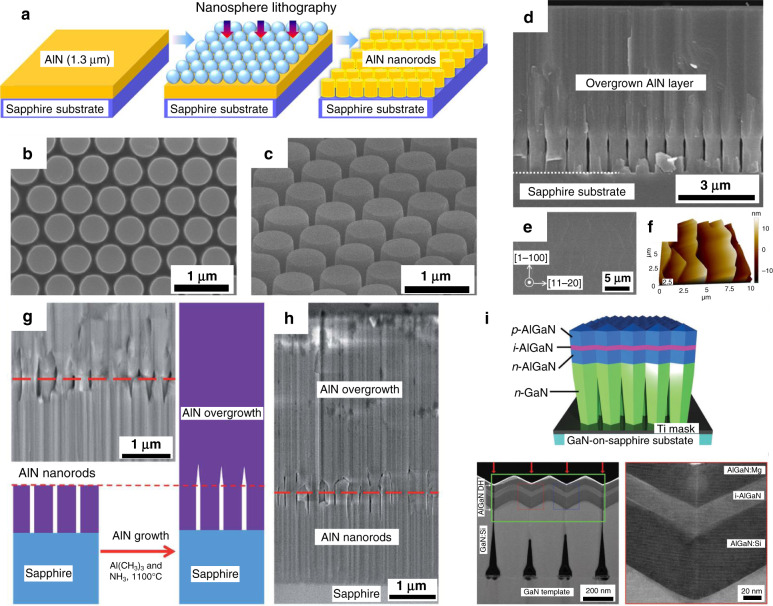
Nanopatterned template for AlGaN/AlN epitaxial lateral overgrowth. **a** Schematic diagram of silica nanosphere lithography. **b**, **c** SEM images of the nano-patterned substrate (NPS). **d** Cross-section and **e** plane-view SEM images of the ELO-AlN layer on the NPS. **f** AFM image shows the macro-steps on the AlN surface. Reprinted with permission from Donghyun et al.^119^. Copyright 2017 American Institute of Physics. **g** SEM cross-sectional image of ELO-AlN nanorod pattern with air gaps. **h** SEM image of ELO-AlN on sapphire. Reprinted with permission from Conroy et al.^121^. Copyright 2015 The Royal Society of Chemistry. **i** Coalesced AlGaN nanowire double heterostructure LED, the following two images are cross-sectional STM-HAADF image and high-magnification image, respectively. Reprinted with permission from Binh et al.^122^. Copyright 2016 WILEY-VCH Verlag GmbH & Co. KGaA, Weinheim

A nanopatterned template can also be used for AlGaN/AlN ELO. In addition to improving crystal quality, the small nanopatterned substrate (NPS) ELO technique requires a thinner coalescence epilayer thickness, which helps reduce the epitaxial manufacturing cost. Donghyun et al.^119^ used nanosphere lithography combined with the ELO method to improve the crystal quality of the AlN epilayer and achieved a small coalescence thickness of below 2 μm. Figure 6a illustrates the silica nanosphere lithography technique. The air voids incorporated by the NPS can effectively relieve the stress of AlN, as shown in Fig. 6b–d. Additionally, the miscut of the sapphire substrate introduces typical zigzag macrosteps^120^, as shown in the AFM image of Fig. 6f. Conroy et al. used self-assembled silica spheres on the surface of AlN to produce the pattern template. Figure 6g, h shows the ELO process and the edge-type dislocation of overgrowth AlN decreases by approximately two orders of magnitude^121^. Le. et al.^122^ demonstrated semipolar AlGaN fabricated by controlled nanowire coalescence and obtained AlGaN quasi 3D film structures with nearly free dislocations (Fig. 6i). Also, based on coalesced film structures, they fabricated UV LEDs and obtained excellent electrical and optical performance. These results unequivocally confirmed the potential applications of the ELO technique in AlGaN-based devices.

Additionally, plenty of other approaches have been used to grow high-crystal-quality AlGaN and AlN, such as modulated V/III ratio growth under HT conditions, migration-enhanced epitaxy, modified epitaxy, and ammonia pulse-flow multilayer epitaxy. Table 1 summarizes the recent progress of AlGaN and AlN epitaxial growth.

**Table 1:** Summary of AlGaN and AlN epitaxial progress

Epitaxial material	Thickness (μm)	Methods	XRD FWHM (arc sec)	Dislocation density (cm^−2^)	Comment	Reference
Al_0.2_Ga_0.8_N	2	GaN buffer	(0002): 570 (20–24): 876	_	Crack network	Chen et al.^107^
		Low temperature (LT)-AlN interlayer with GaN buffer	(0002): 744 (20–24): 1092	6 × 10^9^ (etch pits)	Several cracks	
		LT-AlN and superlattices (SLs) interlayers with GaN buffer	(0002): 726 (20–24): 1014	4 × 10^9^ (etch pits)	Crack free	
		SLs interlayer with GaN buffer	(0002): 384 (20–24): 708	2 × 10^9^ (etch pits)	Crack free	
	1.2	Directly on sapphire	(0002): 876 (20–24): 1398	7 × 10^9^ (etch pits)	Crack free	
Al_0.2_Ga_0.8_N	3	LT AlN and HT Al_0.2_Ga_0.8_N with SLs	(0002): 360 (20–24): 690 for SLs	_	Crack free	Zhang et al.^106^
Al_0.55_Ga_0.45_N	1	PALE and SLs	_	Screw: 7 × 10^7^ Edge: 3 × 10^9^	Dramatically reduce screw-type TD density	Sun et al.^108^
Al_0.2_Ga_0.8_N	0.2	Plastic relaxation and lateral growth	(0002): <340	5 × 10^8^	Crack free	Bethoux et al.^105^
Al_0.4_Ga_0.6_N	0.5	Indium-silicon codoping approach	_	_	Crack free, indium counteract with defect incorporation	Adivarahan et al.^97^
AlN	>10	ELO	(0002): 300 (20–24): 400	<10^7^ (plan-view of TEM)	Crack free	Imura et al.^272^
Al_0.23_Ga_0.77_N	1.5	GaN interlayer	_	2 × 10^8^	Crack free	Jiang et al.^104^
AlN	6	Nanoscaled ELO	_	3.5 × 10^8^	Crack free	Conroy et al.^121^
AlN	8.5	ELO on Si(111) substrate	(0002): 960 (10–12): 810	_	RMS: 0.12 nm	Cicek et al.^118^
Al_0.8_Ga_0.2_N on AlN	Al_0.8_Ga_0.2_N: 3 AlN: 12	ELO-AlN	_	Wing region: <10^7^ Ridge region: 6–9 × 10^8^	Achieve effective defect reduction	Mogilatenko et al.^116^
Al_0.33_Ga_0.67_N on AlN	Al_0.33_Ga_0.67_N: 0.82 AlN: 5	ELO	(0002): 100 (10–12): 500 for AlN	_	Crack free	Kueller et al.^117^
Al_0.8_Ga_0.2_N on AlN	Al_0.8_Ga_0.2_N: 1 AlN: 5	ELO-AlN	(002): 140 (302): 335 for AlN	5 × 10^8^ (plan-view of CL)	0.25° miscut toward a-plane sapphire provide a smooth surface	Zeimer et al.^273^
AlN	5.2	Nanoscaled ELO	(0002): 186 (10–12): 432	_	Crack free	Donghyun et al.^119^
Al_0.1_Ga_0.9_N	3	Nano-patterned sapphire substrate (NPSS) ELO	_	1–2 × 10^9^	Annihilation of TDs is related to stacking faults	Tasi et al.^274^

AlGaN grown on the LT/HT AlN template is a common epitaxial method due to its simple process. However, the two-step method based on the LT/HT AlN template, which includes the LT/MT/HT three-step method, is macroscopically restricted to the level of crack-free thin films in the early research stage. To improve the crystal quality of high-Al-content AlGaN, other epitaxial methods are suggested to be applied simultaneously. Interlayer modulation and SL structure make a significant sense of blocking threading dislocations by releasing stress and strain. Pulse atomic layer epitaxy and pulse flow multilayer growth also contribute to the migration of Al atoms and facilitate the microscopic reduction of threading dislocations. However, concurrently, these methods also proposed higher requirements for epitaxial equipment, which must accurately control the preset gas flux and the thickness of the insert epitaxial layer. The ELO method causes dislocations to bend and annihilate during the epitaxial process. Although this approach is effective, it relies on patterned templates, which inevitably increases the number of epitaxial steps and associated costs. Consequently, various methods can be combined to obtain high-quality AlGaN materials that match different requirements. Currently, the threading dislocation density in AlGaN materials is typically 10^8^ –10^10^ cm^−2^. The potential and application prospects of AlGaN also must be explored in more detail.

In addition to improving crystal quality in the preparation of AlGaN materials, doping is another factor that must be considered. The doping level in PDs with p-n or p-i-n structures also plays a decisive role in device performance. In AlGaN materials, n-type doping has become relatively mature. However, efficient p-type doping still faces severe challenges.

### High-efficiency p-type doping of AlGaN

In III-nitride semiconductors, Mg, Zn, and Be elements are typically used for p-type doping^123–125^, and the corresponding activation energies (AE) of the three elements in GaN are 60, 160, and 370 meV^126,127^. All of their AE increase with the aluminum component in AlGaN alloy. Despite the fact that the AE of Be is lower than that of other elements, it is toxic metal and is more likely to introduce interstitial atoms to compensate acceptors. Therefore, studies commonly use Mg as an impurity acceptor for p-doping of GaN-based materials^128,129^. However, several factors lead to the problems of p-type doping in high-Al-content Al_x_Ga_1-x_N alloys: (i) the low solubility of acceptor dopants in (Al)GaN^130^; (ii) the strong self-compensation effect resulting from the donor-like native defects^131^; and (iii) the high activation energy of the Mg acceptor, which increases from 160 to 500 meV as the Al composition rises (x: 0–1) in Al_x_Ga_1-x_N^128^. The bottleneck problem of p-type doping has long plagued the developing progress of AlGaN devices.

To solve the difficulties of p-type doping of AlGaN alloys, researchers have developed a variety of methods to restrain the self-compensation process, increasing the solubility of Mg and reduce the AE of Mg in AlGaN^77,132,133^. These methods include delta (*δ*) doping, modulation doping, SL doping, codoping, polarization-induced doping, and multidimensional doping^79,96,134–138^.

The delta doping method maintains a steady flux of the group-V source (NH_3_) and alternate Al, Ga, and Mg source supplies so that the Mg source is supplied in an NH_3_ environment^139–142^. Due to the interruption of the group-III source supply, Mg is likely to combine with Al or Ga vacancies, thereby increasing the doping of Mg substitutional atoms in AlGaN, reducing the self-compensation effect, and improving the incorporation efficiency of Mg atoms.

The SL doping method uses energy band engineering to reduce the AE of Mg acceptor impurities^143–149^. In the III-nitride heterostructure, the polarization effect caused by lattice mismatch will generate a polarized electric field, which will cause the energy band near the interface to bend, thereby reducing the acceptor impurities near the interface. It is beneficial to increase the activation efficiency of Mg acceptors and improve the conductivity of p-type materials.

**Fig. 7:**
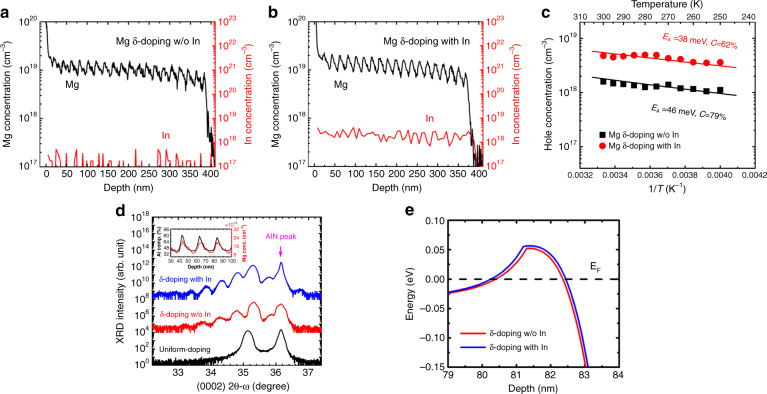
Mg-*δ*-doped AlGaN layers with and without In surfactant. **a**, **b** Mg and In concentration depth profiles. **c** Temperature-dependent hole concentrations. The solid lines are fitting curves. **d** HRXRD (0002) 2θ-ω scan curves. The inset presents the Al-content and Mg concentration depth profiles in the In-surfactant-assisted sample. **e** Calculated self-consistent valence band diagrams at the quantum-well and graded-barrier interface, as a result of Mg-*δ*-doping. Reprinted with permission from Jiang et al.^76^. Copyright 2015 AIP Publishing LLC

Based on the delta doping technique, Jiang et al.^76^ proposed an indium-surfactant-assisted method to achieve a high concentration of holes in Al_0.4_Ga_0.6_N. Figure 7a, b shows the depth profiles of Mg and In concentrations in Mg-*δ*-doped AlGaN layers. With In surfactant assistance, the average Mg concentration was improved from 1.1 × 10^19^ to 1.3 × 10^19^ cm^−3^. Indium desorption produces more vacancies, which promotes Mg occupation and contributes to the incorporation of Mg in the *δ*-doping process.

Figure 7c shows the dependent relation between hole concentration (HC) and temperature. The HC is expressed as the formula

1 $$H = \frac{{1 - C}}{C}\frac{{N_{\mathrm{v}}}}{{\mathrm{g}}}\exp \left( { - \frac{{E_{\mathrm{A}}}}{{kT}}} \right)$$

where *H* is the HC, *C* = *N*_D_*/N*_A_ is the compensation ratio, *N*_v_ = 2(2*πm*_p_^*^kT)^3/2^h^−3^ is the effective valence band state density, *g* is the acceptor degeneracy factor, and *E*_*A*_ is the acceptor activation energy. After fitting the curves in Fig. 7c, the compensation ratio C was found to be obtained of 62% for the In-surfactant sample and 79% for the other sample without the In surfactant. The reduction in the compensation ratio can be attributed to the suppression of nitrogen vacancies (VNs) through the introduction of indium. The activation energy *E*_*A*_ also decreased from 46 to 38 meV with indium surfactant.

As the HDXRD and SIMS (inset) measurements in Fig. 7d show, satellite peaks present the different Al compositions in the epitaxial samples, indicating the self-formed quantum-well (QW) and graded-barrier (GB) heterostructure. Figure 7e presents the calculated valence band diagram of the QW and GB interface. Holes will accumulate at the interface near the Fermi level, thus forming a two-dimensional hole gas (2DHG). In the indium-surfactant-assisted sample, larger interfacial band bending promotes the formation and accumulation of 2DHG holes. Consequently, with enhancement produced by Mg, the suppression of the compensation centers, and the 2DHG incrementation, a high concentration of holes (4.75 × 10^18^ cm^−3^) was achieved along with a low sheet resistivity of 2.46 × 10^4^ Ω/sq for the Al_0.4_Ga_0.6_N epilayer.

**Fig. 8:**
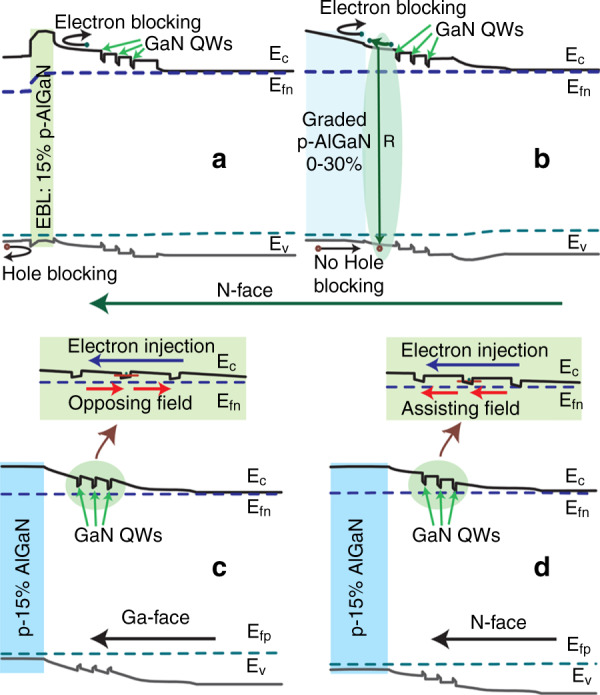
Energy band diagrams of GaN/Al_0.15_Ga_0.85_N MQW. **a** LED of N-polar QW with traditional electron blocking layer and **b** with graded p-AlGaN (polarization-induced p-doping). **c** LED of Ga-face QW: polarization fields oppose electron injection. **d** LED of N-face QW: polarization fields assist carrier injection. Reprinted with permission from Verma et al.^151^. Copyright 2011 American Institute of Physics

Additionally, Simon et al.^150^ proposed an AlGaN heterostructure with graded Al composition and achieved polarization-induced p-type doping of AlGaN for the first time. They grew AlGaN with a graded Al composition on AlN. Because the net charge on the AlN/AlGaN interface induced by polarization is negative, mobile holes can be introduced in the graded AlGaN layer, which is similar to p-type doping. A p-type doped AlGaN alloy with a HC exceeding 2 × 10^18^ cm^−3^ and resistivity of 0.6 Ω cm was achieved. They also employed polarization-induced p-type doping in an N-polar III-nitride QW UV LED^151^. As shown in Fig. 8b, when acting as an electron blocking layer, the polarization-induced p-doped layer removes the energy barriers for holes more effectively than a traditional layer (Fig. 8a). Also, the N-polar structure also facilitates carrier transport in the QWs, as shown in Fig. 8c, d. Thus, polarization-induced p-type doping potentially facilitates higher performance of in AlGaN-based devices.

Other methods have also been reported for improving p-doping efficiency. Kang et al.^137^ proposed a new multidimensional Mg SL doping method to improve the vertical conductivity of AlGaN materials. They calculated the density of states along the [0001] direction of the non-doped and Mg-doped structures via first-principles analysis and found that three-dimensional SL Mg doping reduced the hole barrier and increased the HC in the barrier region, which resulted from the strong *P*_*z*_ hybridization between Mg and N. According to the theoretical results, they prepared an Al_0.63_Ga_0.37_N/Al_0.51_Ga_0.49_N SL structure and obtained high-efficiency p-type doping, with an HC of 3.5 × 10^18^ cm^−3^ and a low resistivity of 0.7 Ω cm. Alternative Mg and Si codoping methods have been also proposed for p-channel device fabrication. Recent results of AlGaN p-type doping are summarized in Table 2.

**Table 2:** Summary methods and results of AlGaN P-type doping

Material	Technique	Hole Concentration (cm^−3^) at RT	Resistivity (Ω cm) at RT	Hole Mobility (cm^2^ V^−1^ s^−1^) at RT	Comment	Reference
Al_0.4_Ga_0.6_N	In-surfactant-assisted Mg-*δ*-doping	4.75 × 10^18^	Sheet Resistivity: 2.46 × 10^4^ (Ω/sq)	1.34	Enhance Mg incorporation, reduce compensation ratio and *E*_A_	Jiang et al.^76^
Graded AlGaN	Polarization-induced doping	1.5 × 10^18^	1	15	Robust to thermal freezeout effects	Simon et al.^150^
Al_0.63_Ga_0.37_N/ Al_0.51_Ga_0.49_N SLs	Multidimensional Mg-doping	3.5 × 10^18^	0.7	2.6	Enhance vertical hole conductivity in Al-rich structure	Kang et al.^136^
Al_0.2_Ga_0.8_N/GaN SLs	Superlattice doping	2.5 × 10^18^	0.2	_	Confined hole gas due to valence band bending	Peter et al.^96^
Graded Al_x_Ga_1-x_N (x: 0 ~ 0.3)	Polarization-induced doping	~10^18^	_	_	Impurity free	Li et al.^275^
Al_0.07_Ga_0.93_N	Mg-*δ*-doping	2 × 10^18^	0.6	5	High vertical conductance, terminate dislocation propagation	Nakarmi et al.^134^
Al_0.7_Ga_0.3_N	V/III ratio modulation doping	2 × 10^17^	47	_	Suppress self-compensation, reduce *E*_A_	Kinoshita et al.^78^
Al_0.5_Ga_0.5_N	Mg-doping	2.2 × 10^17^	10	2.7	Narrow optimal window for Mg doping, excessive Mg incorporation causes defects	Jeon et al.^124^
Al_0.2_Ga_0.8_N/GaN SLs	Modulation-doping	3.4 × 10^18^	0.2	9.2	Reduction of neutral impurity scattering improves mobility	Waldron et al.^276^
AlC on Al_0.8_Ga_0.2_N	Al/C_3_H_8_ treatment of AlGaN template	Sheet carrier density: 10^13^ (cm^−2^)	Sheet Resistivity: 2 × 10^4^ (Ω/sq)	30	AlC as hole injection layer	Kishimoto et al.^277^
Al_0.4_Ga_0.6_N	Alternative codoping	6.3 × 10^18^	0.99	1	Promote development of p-channel devices	Aoyagi et al.^278^
Al_0.1_Ga_0.9_N	C-doping	3.2 × 10^18^	20	0.4	Potential of carbon acceptors in AlGaN	Kawanishi et al.^279^

In our opinion, doping methods can be divided into two types: structure-induced and impurity-intervention. Polarization-induced doping and SL doping use energy-band engineering to modulate holes, thus leading to effective p-type doping. These typical structure-induced doping methods mitigate the demand on activating acceptor impurities. However, these methods can only be applied with specific structures. Impurity-intervention doping is easier to control in epitaxial processes, such as In-surfactant-assisted Mg-*δ*-doping, V/III ratio modulation doping, and alternative codoping. The ultimate goal of these methods is to reduce the activation energy of the acceptor (*E*_A_) and improve doping efficiency. Existing doping methods can increase the HC of AlGaN to 10^18^. Additionally, we must consider the influence of impurity incorporation on the quality of AlGaN alloys. In terms of the subsequent PDs, impurity scattering is also a significant factor that impacts the device performance.

Thus, with the development of epitaxy and doping technology, the crystalline quality and doping level of AlGaN materials are continuously improved, which is conducive to improving the performance of fabricated devices. In addition to the material itself, a variety of PD structures also exhibit great differences in performance, which will be discussed in the next section.

## AlGaN-based solar-blind UV PDs

To date, the research goal of UV PDs has been to obtain devices with low dark current, high responsivity, and large bandwidth. Due to their large and tunable bandgap, good thermal conductivity, high carrier mobility, and superior physical and chemical stability of Al_x_Ga_1-x_N materials, solar-blind UV detection technologies based on Al_x_Ga_1-x_N materials have become a domestic hotspot of extensive research^51,152–155^. Herein, we focus on the structures and properties of the various types of AlGaN solar-blind PDs.

### Photoconductors

Photoconductors have attracted considerable attention for a long time due to their simple fabrication process, high responsivity, low cost merits, and the fact that they can be used in flame and fire monitoring applications^156–158^. The work of photoconductive devices is based on changes in electrical conductivity caused by light excitation. However, due to defects, the photoconductive effect in GaN and AlGaN is typically associated with a slow response speed, low-energy photon response, and severe temperature dependence. Defects in the material trap carriers. Long-term hole trapping causes the minimum hole recombination time to be much higher than the electron transit time, and the charge collection rate is higher than the charge generation rate, which is also the primary reason for the formation of photoconductive gain. The switching speed of the PDs relies on the minority lifetime of the carriers: the longer the minority carrier lifetime is, the higher the gain but also, the lower the switching speed^159–163^. Therefore, a trade-off between gain and speed must be considered. Additionally, the existence of defect energy levels can also cause a low-energy photon response. The defect-related carrier capture and release mechanism is also fed back to the frequency characteristics of the device. For example, the out-of-band response is higher at low frequencies.

The first GaN-based PD implemented by Khan et al.^164^ is a photoconductive detector. Only two metals must be deposited onto the GaN film for ohmic contact. However, the shortcomings of this PD also include a slow response speed and large leakage currents. In addition, solar-blind detection requires using AlGaN materials.

**Fig. 9:**
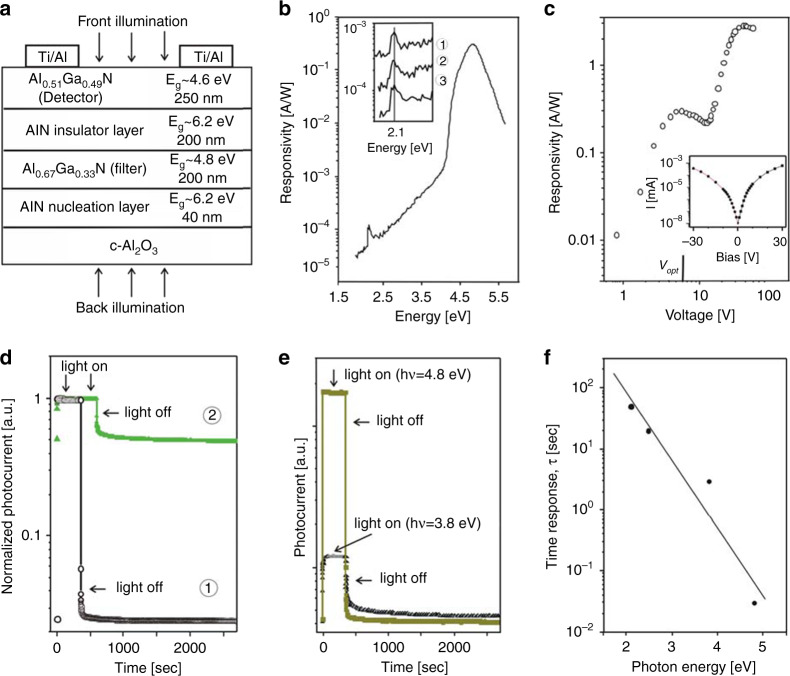
Structure and performance of an AlGaN solar-blind photoconductor. **a** Schematic structure of AlGaN photodetector. **b** Spectral responsivity of the AlGaN detector at back-illumination under 5 V bias. The insert illustrates the photocurrent resonance peak, measured at 5 V (curve 1), 12 V (curve 2), and 15 V (curve 3), respectively. **c** Spectral responsivity versus applied voltage (*hν* ~ 4.8 eV). The inset presents the dark current. *V*_opt_ is the trap-free point (6 V). **d** Time response (*hν* ~ 4.8 eV) at 5 V (dark circles) and 15 V (green triangles). **e** Time response at 5 V (trap-free regime) with 4.8 eV photon (squares) and 3.8 eV photon (triangles). **f** Time constant versus photon energy at 5 V. Reprinted with permission from Cherkashinin et al.^55^. Copyright 2006 WILEY-VCH Verlag GmbH & Co. KGaA, Weinheim

In 2004, Lebedev et al.^157^ fabricated a photoconductive solar-blind Al_0.51_Ga_0.49_N detector with an Al_0.67_Ga_0.33_N integrated filter and obtained a high solar-blind responsivity with a narrow wavelength range, verifying the functionality of the filter. They also confirmed that defect states will lead to subband and near-bandgap absorption in AlGaN optoelectronic detectors. In 2006, they also studied the responsivity and time response of AlGaN solar-blind photoconductors. Figure 9a shows the schematic structure of the AlGaN detector. As shown in Fig. 9b, the PD achieves a spectral detection range from 220 to 300 nm. Additionally, the photocurrent peaks in the inset of Fig. 9b can be attributed to the transition between the localized deep acceptor state and shallow donor state^152^. Notably, there are two response shoulders in Fig. 9c, indicating the effects of traps on the photoconductivity. The onset drop of the photoresponse at *V*_opt_ (6 V) can be attributed to excess trapped carrier density. At higher voltage, the free carrier density is sufficiently large to neglect the trapping effect. Also, the effect of trapping on the photoconductivity increases the decay time. Figure 9d shows the time response characteristics of the PD at low (5 V) and high (15 V) voltages. With a 15-V bias, the device exhibits a significant defect-related persistence photoconductivity (PCC) effect. Conversely, when the bias is lower than 7 V, no marked PCC effects are observed. The characteristic time constant (*τ*) can be extracted from the following expression:

2 $$I\left( t \right) = I_0 + B_0\exp \left[ { - \left( {\frac{t}{\tau }} \right)^\beta } \right]$$

where *I*_0_ is the preliminary dark current, *B*_0_ is the exponential term, and *β* is the decay exponent. For the 5-V and 15-V curves, *τ* is 0.03 and 0.7 s, respectively. However, PCC effects also occur in the trap-free regime (bias <7 V) when the photon energy is 3.8 eV, as shown in Fig. 9e. The time constant *τ* increases with decreasing photon energy (see Fig. 9f) and becomes independent at the lower bias value (<6 V). They concluded that there is an optimal parameter (6 V) to achieve a compromise between the response time and spectral responsivity. The trapping of space charges accounts for the increase of decay time.

### Schottky barrier photodiodes

The structure and fabrication process of AlGaN-based Schottky detectors are relatively simple and consist of a Schottky contact metal and an ohmic contact metal. The photoelectric response speed is fast, and the response time is on the order of ns. But the response speed is restricted by the RC time constant. Among the various available detectors, the Schottky detector has the widest flat-band response window in a short wavelength region, which is suitable for manufacturing detectors and arrays. Its responsivity is near 0.1 A/W at zero bias^165,166^ and the response ratio of UV to visible light is typically in the range of 10^3^ to 10^4^.

In 1998, Osinsky et al.^167^ reported the first AlGaN Schottky solar-blind UV photodiode, which exhibits a response of 70 mA/W at 272 nm at 0 V, with an external quantum efficiency (EQE) of 32%. The fabricated AlGaN Schottky detector exhibits superior solar-blind characteristics, and the UV/visible rejection ratio reaches 10^4^.

Miyake et al. proposed front-illuminated solar-blind AlGaN Schottky PDs on a large scale^168^. The fabricated devices operated at wavelengths of 100–265 nm. The UV/visible rejection ratio is ~10^4^. The manufactured AlGaN-based solar-blind detectors could be employed for the VUV detection. Additionally, Biyikli et al. produced a low-noise and high-detectivity Schottky photodiode for solar-blind UV detection^169^. AlGaN/GaN heterostructures were used to achieve better ohmic contact. The cutoff wavelength was located at 274 nm, corresponding to the Al content of AlGaN absorption layer. The proposed devices exhibited a low dark current density of 1.8 nA/cm^2^ in the bias range of 0–25 V. The PD exhibited a maximum EQE of 42% at 267 nm. Moreover, the device exhibited a high detectivity, which exceeded 2.6 × 10^12^ cm Hz^1/2^ W^−1^, and a low noise power density below 3 × 10^−29^ A^2^/Hz at 10 kHz.

**Fig. 10:**
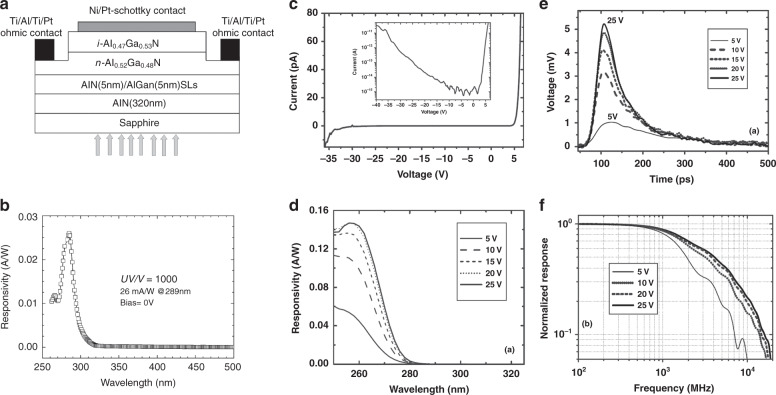
Structure and performance of AlGaN Schottky barrier photodiodes. **a** Schematic structure of Schottky AlGaN photodetector. **b** Responsivity of the fabricated device at 0 V. Reprinted with permission from Sang et al.^166^. Copyright 2008 Chinese Physical Society and IOP Publishing Ltd. **c** Dark current of the fabricated AlGaN photodetector. **d** Responsivity of the devices at different applied voltages. **e** Pulse response of the devices at different applied voltages. **f** Corresponding fast Fourier transform curves. Reprinted with permission from Tut et al.^169^. Copyright 2004 Elsevier Ltd

Sang et al.^166^ proposed an AlGaN-based solar-blind PD (Fig. 10a, b) with back illumination and used Ni/Pt Schottky contacts to reduce the leakage current. Figure 10b shows that the device exhibits a significant peak at 289 nm. One of the major merits of Schottky detectors is their fast response speed. Biyikli also proposed an indium-tin-oxide (ITO) Schottky PD with a high response speed (3-dB bandwidth: 1.10 GHz). Also, high bandwidth-efficiency AlGaN Schottky photodiodes were demonstrated by Tut et al.^169^. As shown in Fig. 10c, the fabricated device produced an extremely low dark current at the level of 3 fA when the applied voltage was lower than 12 V. The solar-blind cutoff edge was ~266 nm, as shown in Fig. 10d. At an applied voltage of 20 V, they obtained a peak responsivity of 147 mA/W at 256 nm with rejection ratio in excess of four orders of magnitude. Also, the device with a diameter of 30 μm achieved a 53-ps pulse-width and a 4.1-GHz BW, which are extracted from Fig. 10e, f. The fabricated AlGaN Schottky photodiode obtained a BW-efficient product of 2.9 GHz, demonstrating the device’s superior response speed performance.

### Schottky metal–semiconductor–metal PDs

A metal–semiconductor–metal (MSM) PD is composed of two back-to-back Schottky diodes and has the advantages of a simple structure and manufacturing process, a low dark current, a fast response, and an easy planar integration. The RC time constant can be reduced by controlling the electrode spacing of the interdigital structure, which is suitable for high-speed photoelectric conversion applications^170–174^.

In 1997, Carrano et al.^175^ reported that a Schottky GaN UV detector with an MSM structure had a dark current of 57 pA at 10 V and achieved a 0.4 A/W responsivity under a bias of 6 V. In 1999, Monroy et al.^176^ reported n-type and p-type MSM structure GaN UV detectors with response times of 10 and 200 ns, respectively. In 2004, Li et al.^177^ reported an MSM-structured GaN UV PD with a response time of 4.9 ps. Several research groups have reported on AlGaN solar-blind UV detectors with front- or back- illuminated MSM structures^178–181^.

**Fig. 11:**
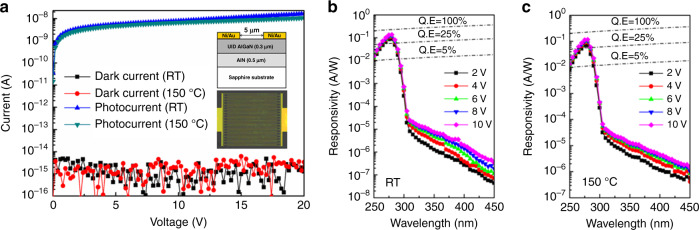
Structure and performance of a Schottky metal-semiconductor-metal photodetector. **a** I-V curves of the MSM detectors measured in dark and under 254 nm UV illumination at RT and 150 °C conditions, respectively. Spectral response of the devices measured at **b** RT and **c** 150 °C. Reprinted with permission from Xie et al.^182^. Copyright 2012 IEEE

Xie et al. fabricated an AlGaN MSM solar-blind UV detector with an ultralow dark current based on HT-AlN epitaxy^182^. At room temperature and at 150 °C, its dark current remains in the fA order of magnitude, as shown in Fig. 11a. The device has a room temperature EQE of 64% (at 275 nm) at applied voltage of 10 V, and the solar-blind UV/near UV rejection ratio reaches 10^4^ (Fig. 11b). The EQE of the device remains above 50% even at 150 °C, and the UV/visible rejection ratio exceeds 8000, as shown in Fig. 11c. These results also confirmed that the AlGaN solar-blind detectors can withstand higher operating temperatures.

**Fig. 12:**
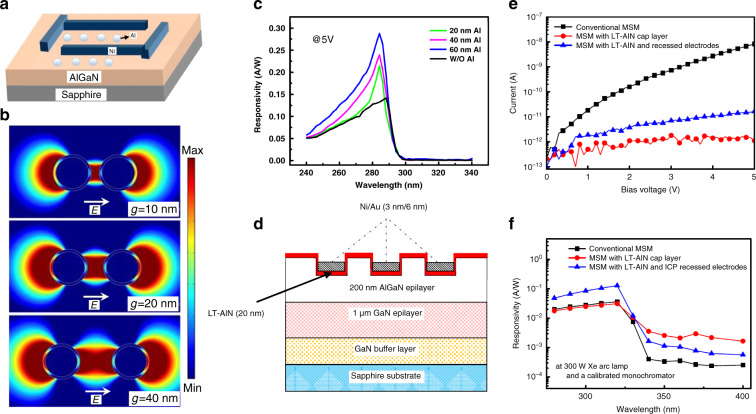
Performance improvement methods on AlGaN MSM photodetectors. **a** Schematic structure of AlGaN MSM photodetector with Al nanoparticles. **b** Electric field distribution of Al nanoparticles with changing gaps. **c** Responsivity of devices with and without Al nanoparticles. Reprinted with permission from Li et al.^187^. Copyright 2014 Optical Society of America. **d** Schematic structure of AlGaN with low-temperature AlN cap layer and recessed electrodes. **e** Dark current curves of the three samples. **f** Spectral response of the three samples. Reprinted with permission from Chen et al.^188^. Copyright 2010 The Japan Society of Applied Physics

Using the MSM structure, researchers have proposed various methods to increase device performance^183–185^. Nanoplasmonic enhancement can be employed on the solar-blind photodetector^186^. Li et al.^187^ demonstrated that Al nanoparticles enhanced the responsivity of an AlGaN MSM solar-blind detector, as shown in Fig. 12a, c. By investigating the electric field distribution of Al nanoparticles (seen in Fig. 12b), they found that the localized surface plasmon resonance effects contributed to the enhancement in responsivity of the MSM detectors. Chen et al.^188^ also fabricated an MSM AlGaN PD with a low-temperature (LT) AlN layer and recessed electrode structure, as shown in Fig. 12d. The improved structure shows a lower dark current than the conventional structure in Fig. 12e. Highly resistive LT-AlN provides a higher potential barrier. Figure 12f shows that the LT-AlN and a recessed electrode structure yield a higher responsivity, which can be ascribed to the enhanced electric field intensity between electrodes and higher photoconductive gain. Additionally, polarization engineering can also be used to enhance the performance of AlGaN solar-blind detectors by introducing high electron mobility conduction channel^189,190^.

### p-n, p-i-n photodiodes

Most p-n or p-i-n junction detectors exhibit have many advantages, including a low working bias, a high input impedance, a high working frequency, and an integration capability that is useful for manufacturing technologies and semiconductor planar processes^191–194^. Unlike the p-n junction, the width of the space charge region in the p-i-n structure does not depend on the p-n junction electric field, but is primarily determined by the thickness of the i-type layer. Therefore, the design of the thickness in an unintentionally-doped (i-type) layer is important. A thick i-type layer can ensure sufficient light absorption to improve the quantum efficiency of the detector, which facilitates reducing the junction capacitance and the RC time constant. However, this layer will concurrently increase the transit time of photogenerated carriers and reduce the response speed of the detector. Hence, it is necessary to compromise the design according to demands of real applications.

**Fig. 13:**
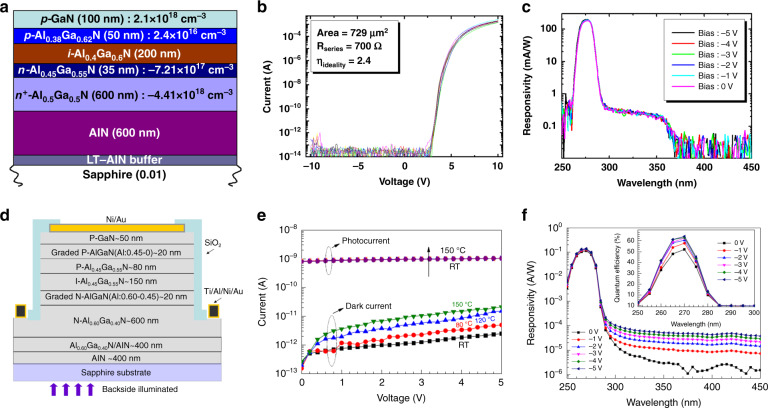
Structures and performance of AlGaN p-i-n photodiodes. **a** Schematic cross-sectional structure of p-i-n AlGaN photodetector. **b** I-V curves of ten devices on the same wafer. Turn-on voltage is 5.6 V. The label shows series resistance and ideality factor. **c** Responsivity at different reverse biases. 176 mA/W under zero bias and 192 mA/W at 5 V. Reprinted with permission from Cicek et al.^199^. Copyright 2013 AIP Publishing LLC. **d** Schematic cross-sectional structure of the photodetector. **e** I-V curves at different temperatures. **f** Responsivity at various biases. The inset illustrates quantum efficiency in the wavelength range from 250 to 300 nm. Reprinted with permission from Wang et al.^200^. Copyright 2012 Chinese Physical Society and IOP Publishing Ltd

In 1999, Parish et al.^195^ prepared a p-i-n structure AlGaN solar-blind UV detector on a laterally epitaxial GaN template. Its peak response was 0.05 A/W at 285 nm, and the dark current density was 10 nA/cm^2^, and the response time was extremely low (4.5 ns). Biyikli et al.^196^ employed a recessed etching process on the p^+^-GaN cap layer in a p-i-n structure AlGaN solar-blind UV detector. The dark current of the fabricated device was as low as 3 fA under a 6-V bias, and the detectivity reached 4.9 × 10^14^ cm Hz^1/2^ W^−1^. Collins et al.^197^ used high-Al-content n-Al_0.6_Ga_0.4_N as the optical window layer in the p-i-n structure AlGaN detector to enhance the light transmission to the AlGaN solar-blind absorption region, and obtained a detectivity up to 2.0 × 10^14^ cm Hz^1/2^ W^−1^. Researchers at Northwestern University adopted a high-Al-content p-Al_0.7_Ga_0.3_N as the optical window layer in AlGaN p-i-n detector and obtained a response peak (at 262 nm) of 0.20 A/W at zero bias. The UV/visible rejection ratio reached 10^6^ ^198^. In 2013, Cicek et al.^199^ used a Si-In codoped Al_0.5_Ga_0.5_N window layer and a high-quality AlN template to prepare a back-illuminated p-i-n structure AlGaN solar-blind UV detector, as shown in Fig. 13a–c. An EQE of 80% at 275 nm was obtained at zero bias, and an EQE of 89% was achieved at a 5-V applied voltage. The UV/visible light rejection ratio exceeded six orders of magnitude. Multisample measurements verified the uniformity of the device performance.

When manufacturing AlGaN solar-blind UV detectors, HT-AlN buffer layers and AlN/AlGaN SL structures are often used to suppress the accumulation of tensile strain in the epitaxial process of the AlGaN layer and reduce the dislocation density of the AlGaN layer. The research team of Nanjing University used this method to prepare a p-i-n structure AlGaN solar-blind UV detector^200^, as shown in Fig. 13d–f. The leakage current was as low as 1.8 pA, and the peak quantum efficiency reached 64%. The temperature dependent I-V curve shows that the dark current increases marginally with increasing temperature, which can be attributed to defect-related parasitic leakage^201^. However, the dark current remains on the order of 10 pA. The thermal-noise-limited detectivity reaches 3.3 × 10^13^ cm Hz^1/2^ W^−1^.

### Avalanche photodiodes

APDs can obtain gain via the impact ionization process of carriers under breakdown conditions so that they can detect weak UV signals^156,202–206^. The APD exhibits two modes: linear and Geiger. When avalanche breakdown occurs and the device achieves a large multiplication gain, the device works in Geiger mode, which plays a significant role in the field of single-photon detection^207–211^. The multiplication factor can be extracted from the following formula

3 $$M = \frac{1}{{1 - {\int}_0^L {\alpha \left( x \right)dx} }}$$

where L is the movement length and *α* is the multiplication coefficient of carriers. The impact ionization coefficient is related to the material itself (Al content in Al_x_Ga_1-x_N) and to external factors such as electric field intensity, temperature, etc. Experimentally, the relationship between the multiplication gain and current–voltage can be expressed as

4 $$G = \frac{{I_{\mathrm{M}}^{{\mathrm{light}}} - I_{\mathrm{M}}^{{\mathrm{dark}}}}}{{I_0^{{\mathrm{light}}} - I_0^{{\mathrm{dark}}}}}$$

where *I*_M_ and *I*_0_ are the multiplied and unmultiplied currents, respectively^203,212,213^.

The APD that has been reported so far includes the various structures discussed above, including Schottky, p-n, and p-i-n structures. In particular, a separate absorption and multiplication (SAM) structure has also been proposed to enhance device performance. In the absorption region of the SAM structure, the photogenerated carriers are separated by the bias voltage. The single type of carrier is accelerated into the multiplication region through the electric field, thereby causing massive impact ionization and triggering avalanche events. This single-carrier triggering avalanche mechanism can reduce the excess noise of the device^214,215^. Considering the materials used in APDs, high-Al-content AlGaN APDs are typically prepared on AlN/sapphire templates, and the material quality and performance of the fabricated devices are better than those used on GaN templates.

Currently, UV GaN APDs^216,217^ have made great progress and achieved single photon detection with multiplication gain exceeding 10^5^. However, AlGaN-based solar-blind APDs are rarely reported^218–221^ and the development of the AlGaN APD marginally lags behind that of GaN. The primary reasons for the slow development of AlGaN APDs include difficulties in achieving high-quality material epitaxy, the problem of obtaining high-efficiency p-type doping, and the change in impact ionization coefficient with Al composition in Al_x_Ga_1-x_N alloys^222,223^.

A solar-blind AlGaN APD with a maximum gain of 700 was reported by McClintock et al.^218^. Under a low bias voltage, the device exhibits soft breakdown, and Geiger operating mode does not occur. Tut et al. proposed a Schottky solar-blind APD that achieved an avalanche gain of 1560 after repeatability measurements. They obtained a high thermally limited detectivity of 1.4 × 10^14^ cm Hz^1/2^ W^−1^ ^219^. To produce high-quality AlGaN materials for avalanche device fabrication, Sun et al. inserted an Al_0.4_Ga_0.6_N/AlN SL structure with six periods into the interface between the p-i-n active layer and HT-AlN buffer layer. The employment of an SL structure effectively relieved the strain and reduced the dislocation density^220^. Through the SL modulating epitaxial method, the manufactured AlGaN APD achieved a high gain of 2500 at 62 V applied voltage. The temperature-dependent dark current characteristics verified that APD has a positive temperature coefficient, and breakdown is caused by avalanches, rather than tunneling and photoconductive gain related to defects.

**Fig. 14:**
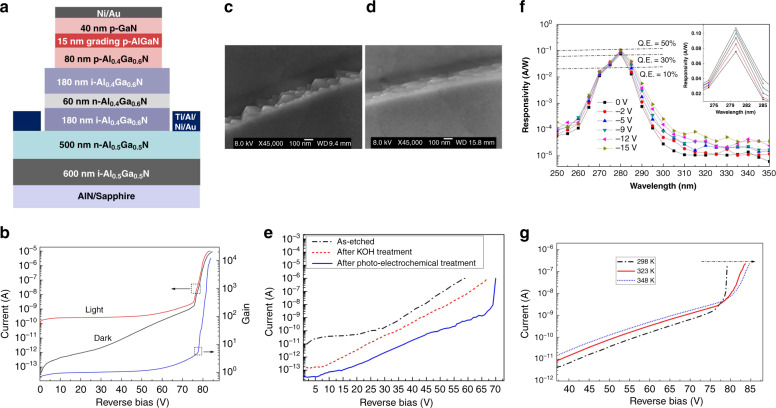
Structure and performance of an AlGaN SAM APD. **a** Schematic cross-sectional diagram of the SAM AlGaN APD. **b** I-V curve in dark and under illumination. Sidewall SEM images of APD with KOH (**c**) and photo-electrochemical (**d**) treatment, respectively. **e** Dark currents for samples with different treatments. **f** Spectral response at various applied voltages. **g** Dark currents at various temperatures. Reprinted with permission from Shao et al.^224^. Copyright 2014 IEEE

Because the gain of AlGaN APD with a conventional p-i-n structure is limited at the magnitude of 10^3^, Shao et al. propose the back-illuminated SAM structure, as shown in Fig. 14a. By employing the SAM structure, nearly pure holes can be injected from absorption region into the multiplication region^224^. The higher hole ionization coefficients contribute to the larger multiplication gain. The SAM APD exhibits a significant avalanche breakdown characteristic at the applied voltage of 75.5 V (Fig. 14b). Furthermore, they developed a photoelectrochemical treatment process to repair etching-induced damage, and the surface defects were effectively passivated. The SEM images show that the photoelectrochemical treatment can remove or smooth whiskers and pyramids on the sidewall of the fabricated APDs induced by ICP dry-etching, as shown in Fig. 14c, d. The optimized flatter sidewall can reduce the risk of local breakdown or premature microplasma breakdown, which can result from a high local electric field formed at the cusp of whiskers and pyramids. The current–voltage characteristics of the fabricated AlGaN SAM APD showed that the photoelectrochemical treatment can effectively reduce the leakage current and improve the yield and gain of APD devices, as shown in Fig. 14e. Combining this optimized method, the device obtained a high gain of 1.2 × 10^4^ at 84-V bias, as shown in Fig. 14b. The response peak located at 280 nm can be observed in Fig. 14f. According to the current–temperature characteristics of the device, as shown in Fig. 14g, a positive temperature coefficient indicates that the source of the gain is avalanche multiplication.

**Fig. 15:**
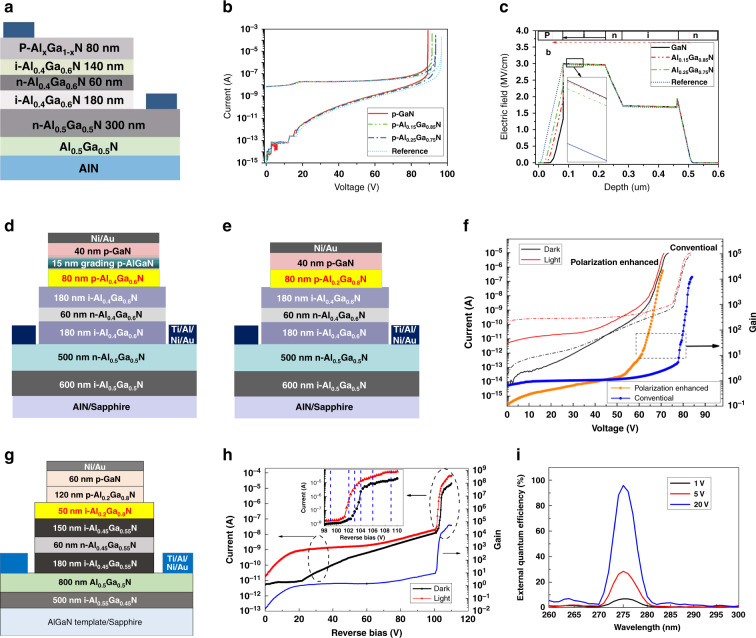
Polarization engineering for improving device performance. Schematic structures of various back-illuminated SAM APDs: **a** simulated polarization-enhanced, **d**, **e** conventional counterpart and experimental polarization-enhanced, **g** ionization-enhanced. **b**, **f**, **h** The I-V and gain curves of the proposed APDs, respectively, transverse corresponding. **c** Electric field distribution of sample a. **i** EQE of sample **g**. Reprinted with permission from Dong et al.^225^ and Shao et al.^227,228^. Copyright 2013 IEEE, 2014 IEEE and 2017 IEEE

To further improve device performance, Dong et al. exploited polarization engineering in an AlGaN SAM APD^225^, as shown in Fig. 15a. By adjusting the Al composition of the p-AlGaN layer, the polarization field and doping effect can be introduced into the APDs. The spontaneous and piezoelectric polarization in GaN-based semiconductors can introduce an internal electric field up to several MV/cm, which is of the same order of magnitude as the avalanche breakdown electric field in the multiplication region of AlGaN APDs. Therefore, by adjusting the Al content of AlGaN, a polarization electric field with the same direction as the reverse bias can be introduced into the multiplication region of the APDs (Fig. 15c). Figure 15b shows that the polarization-induced electric field can significantly reduce the avalanche breakdown voltage. The maximum multiplication gain is also pronouncedly enhanced by the polarization enhancement. Bulmer et al. calculated that the reasonable design of AlGaN APDs with polarization engineering could reduce operating avalanche voltage by nearly 40%^226^. Experimentally, Shao et al. manufactured a polarization-enhanced AlGaN SAM APD by reducing the Al component of the p-AlGaN layer, as shown in Fig. 15e. Figure 15d presents a conventional APD referenced counterpart^227^. The polarization-enhanced APD presents a markedly lower avalanche breakdown voltage and significant higher avalanche gain of 2.1 × 10^4^ compared to the counterpart, as shown in Fig. 15f. The dark current also decreases significantly by one order of magnitude at the onset of breakdown.

Additionally, Shao et al. proposed and fabricated an AlGaN heterostructure APD with high/low-Al-content AlGaN layers as the multiplication region rather than a conventional homogeneous AlGaN layer based on the SAM structure, as shown in Fig. 15g^228^. The carrier multiplication process is primarily initiated by impact ionization of holes, and the design of the Al_0.2_Ga_0.8_N/Al_0.45_Ga_0.55_N heterostructure in the multiplication region can induce a band offset to facilitate hole ionization. The holes can obtain additional energy from a valence band offset, and suppress the electron ionization benefitting from the barrier generated at the conduction band. Thus, the designed heterostructure is expected to reduce the excess noise of APDs, which can be attributed to the electron-initiated multiplication process. Also, the low-Al-content Al_0.2_Ga_0.8_N layer can increase the average hole impact ionization coefficient across the entire multiplication region and achieve a higher avalanche gain. Consequently, they obtained a maximum gain of 5.5 × 10^4^ and a solar-blind response peak at 275 nm (Fig. 15h, i).

In addition to basic device structures, fabrication techniques can also be employed to improve device performance^210^. For example, bevel mesa and field plate terminal technologies can effectively homogenize the electric field distribution and are conducive to reducing the dark current and prevent the early breakdown of the device. The recessed electrode structure is also able to facilitate electric field enhancement.

Polarization field control and energy-band engineering have become increasingly important means for the design of AlGaN heterostructure PDs. Strong polarization is a unique characteristic of nitride semiconductor systems. The polarization field has a strong modulation effect on the energy band structure of the heterojunction, which will significantly affect device performance. Polarization-induced energy band engineering is important in device structure design. How to use the polarization field to enhance device performance or avoid the negative effects of the polarization field is critical to describe and must be considered in the structural design of nitride semiconductor devices. To review the progress of AlGaN-based solar-blind PDs comprehensively, we summarized the characteristics of the reported devices in Table 3.

**Table 3:** Summary of the reported parameters of various AlGaN-based solar-blind photodetectors

Material	Structure	Dark current (A)	Responsivity (A/W)	Rejection ratio	Detectivity (cm Hz^1/2^ W^−1^)	Gain	Reference
Al_0.51_Ga_0.49_N	photoconductor	10^−7^@30 V	0.3@258 nm	~10^3^	_	_	^55^
Al_0.26_Ga_0.74_N	Schottky	10^−9^	0.07@272 nm	~10^4^	_	_	^167^
Al_0.5_Ga_0.5_N	Schottky	10^−7^@-10 V	0.033@206 nm	~10^4^	_	_	^168^
AlGaN/GaN	Schottky	1.5 × 10^−13^@0 V	0.09@267 nm	~10^4^	2.6 × 10^12^	_	^169^
Al_0.38_Ga_0.62_N/GaN	Schottky	3 × 10^−15^@-12 V	0.147@256 nm	>10^4^	1.8 × 10^13^	_	^165^
Al_0.4_Ga_0.6_N	MSM	10^−15^@20 V	0.143@275 nm	~10^4^	_	_	^182^
Al_0.6_Ga_0.4_N/Al_0.5_Ga_0.5_N	MSM	10^−11^@5 V	10^6^@220 nm	~10^6^	_	_	^280^
Al_0.6_Ga_0.4_N	MSM	<2 × 10^−9^@10 V	0.51@230 nm	~10^2^	_	_	^281^
Al_0.4_Ga_0.6_N	MSM	10^−12^@30 V	0.14@275 nm	~10^4^	_	_	^282^
Al_0.5_Ga_0.5_N	MSM	3.32 × 10^−6^@20 V	0.2@251 nm	>10^3^	_	_	^283^
Al_0.33_Ga_0.67_N/GaN	p-i-n	10^−8^ A/cm^2^@-5 V	0.05@286 nm	~10^4^	_	_	^195^
Al_0.45_Ga_0.55_N	p-i-n	<3 × 10^−15^@-6 V	0.11@261 nm	~10^4^	4.9 × 10^14^	_	^196^
Al_0.48_Ga_0.52_N/Al_0.57_Ga_0.43_N	p-i-n	8.2 × 10^−11^ A/cm^2^@-5 V	0.09@269 nm	~10^3^	2.0 × 10^14^	_	^197^
Al_0.36_Ga_0.64_N/Al_0.45_Ga_0.55_N	p-i-n	2.3 × 10^−4^ A/cm^2^@-5 V	0.136@282 nm	~10^3^	_	_	^198^
Al_0.45_Ga_0.55_N	p-i-n	1.8 × 10^−12^@-5 V	0.114@270 nm	~10^4^	3.3 × 10^13^	_	^200^
Al_0.38_Ga_0.62_N	p-i-n avalanche	1.6 × 10^−8^ A/cm^2^@0 V	_	_	_	700	^218^
Al_0.4_Ga_0.6_N	p-i-n avalanche	<8 × 10^−15^@-20 V	0.13@272 nm	~10^4^	1.4 × 10^14^	1560	^219^
Al_0.4_Ga_0.6_N	p-i-n avalanche	3.3 × 10^−12^@-10 V	0.08@270 nm	_	_	2500	^220^
Al_0.38_Ga_0.62_N	SAM avalanche	1.0 × 10^−8^ A/cm^2^@-20 V	0.132@281 nm	~10^3^	_	3000	^221^
Al_0.4_Ga_0.6_N	SAM avalanche	10^−13^@-15 V	0.15@280 nm	~10^4^	_	1.2 × 10^4^	^224^
Al_0.4_Ga_0.6_N	SAM avalanche	5 × 10^−10^@-60 V	_	_	_	2.1 × 10^4^	^227^
Al_0.2_Ga_0.8_N/Al_0.45_Ga_0.55_N	SAM avalanche	~10^−11^@-20 V	EQE 95.8%@275 nm	_	_	5.5 × 10^4^	^228^
Al_0.65_Ga_0.35_N	p-i-n avalanche	~10^−13^@-75 V	0.06@255 nm	>1.2 × 10^4^	_	10^5^	^284^

With the development of fabrication techniques, the structures of AlGaN devices have become more diversified and achieve different performances in various application scenarios. Although photoconductive devices have a simple fabrication process, the signal recognition rate is low due to the high dark current. Using the Schottky structure, the dark current can be reduced by several orders of magnitude. The solar-blind UV/visible rejection ratio can reach 10^4^, and the back-to-back Schottky MSM structure can promote the extraction of photogenerated carriers. In addition to realizing AlGaN solar-blind UV detection, this structure also has the advantage of a simple preparation technique (typically one-step lithography). The planar structure of the AlGaN PD can also be combined with the nanoplasmonic enhancement method to improve responsivity. However, the uneven distribution of the electric field results in the effective detection area of the planar structure being restricted to the device surface. Traps such as surface states will lead to unexpected persistent photoconductance effects, thus reducing the response speed of the device. A vertical p-i-n structure is proposed to achieve a detectivity of 10^14^ cm Hz^1/2^ W^−1^. Thus, an avalanche PD is developed that can detect weak light signals with its large multiplication gain. The existing gain of AlGaN PDs can reach 10^5^, which encourages the research of single photon detection in solar-blind UV light.

However, due to epitaxial problems in high-Al-content AlGaN materials, the threading dislocations of AlGaN typically exceed 10^8^ cm^−2^. There remains a long pathway to realize solar-blind UV single photon avalanche diodes (SPADs). There is also a lack of experimental extraction of impact ionization coefficients in AlGaN. Furthermore, the physical mechanisms of different dislocations on the leakage currents and the influences of impurity scattering as well as the capture of carriers by defects on device performance remain to be studied. Despite these gaps in the literature, the substantial progress has been made in the development of individual devices, which provides support for FPAs and opens a new era in AlGaN solar-blind UV imaging.

## Focal plane arrays

FPA imaging is an important application of UV detection and has promoted significant revolutions in imaging technology. FPA must be combined with a readout integrated circuit (ROIC) to complete signal detection^229,230^. The silicon-based integrated circuit is typically used as a bridge for detection. Thus, the development of the FPA matches the development of integrated circuits (i.e., Moore’s law). FPA imaging has made considerable progress in long wavelength bands such as infrared, far-infrared (terahertz), and sub-mm wavelengths^231–240^. With the development of GaN-based materials, in recent decades, the application of FPAs in the field of UV and deep UV imaging has become more extensive^241–243^.

### FPA architectures

Typically, FPAs are divided into monolithic-integration and hybrid-integration architectures. Additionally, in terms of application types, FPAs can be classified as PDs or thermal detectors^244–248^. We focus on solar-blind UV FPAs in this review.

In monolithic-integration architectures, both optical detection and signal readout (multiplexing) are completed in the same detection material. This approach can reduce the number of process steps, reduce preparation costs, and increase yields. CCD and CMOS sensors are two commonly used types of image sensors, both of which use photodiodes for photoelectric conversion of light into digital data. The primary difference in how they operate is the way they transmit digital data. The charge data of each pixel in each row of a CCD sensor is transferred to the next pixel in sequence, output from the bottom of the pixel, and then amplified and output by the amplifier at the edge of the sensor. In a CMOS sensor, each pixel is adjacent to an amplifier and an A/D conversion circuit, which outputs data in the same manner as a memory circuit. The reason for this difference is as follows: (i) CCDs’ mechanism ensures that the data will not be distorted during transmission. Thus, the data of each pixel can be gathered at the edge of the sensor and then amplified; (ii) CMOS data exhibits more noise when transmitted over longer distances and thus must be amplified first, followed by integrating the data from each pixel. CCD sensors are superior to CMOS sensors in terms of sensitivity, resolution, and noise control, while CMOS sensors are low cost, require little power, and can be highly integrated. However, with the advancement of CCD and CMOS sensor technologies, the differences between the two are gradually shrinking. For example, CCD sensors are starting to use less power for applications in the mobile communications market (e.g., Sanyo), and CMOS sensors are starting to achieve higher resolutions and sensitivities for use in upscale imaging products.

**Fig. 16:**
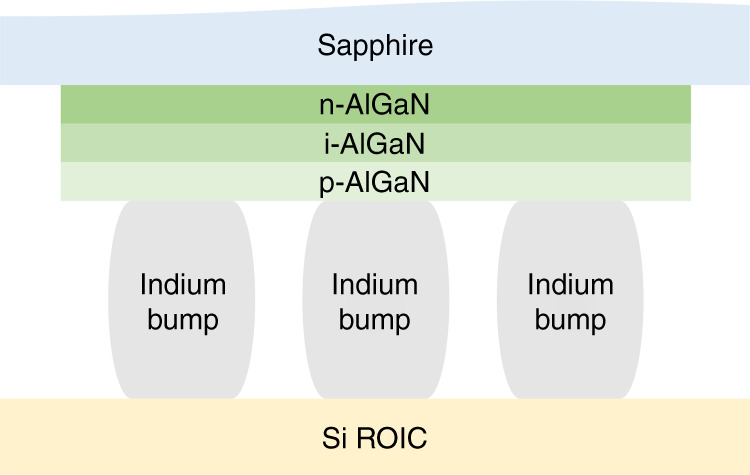
Hybrid-packaged AlGaN focal plane arrays. Indium bump for FPA interconnect AlGaN solar-blind UV photodetector with silicon ROIC

In terms of the hybrid-packaged structure, the detector and multiplexer are optimized independently. UV solid-state imagers are primarily manufactured with a hybrid structure. The merits of the hybrid structure include nearly 100% fill factors and a large area for processing signals on the multiplexer chip. PDs are interconnected with ROIC to describe signal reading. In 1977, hybrid packaging technology was developed and used for production in the following decades. The most commonly used hybridization approach is flip-chip interconnected by bump bonds (indium), as shown in Fig. 16. The detector array is integrated with the silicon readout circuit through the indium bump. Heating causes the indium bump to melt, and the soldering process is implemented by reflow^249–251^.

In addition to the indium bump technique, other approaches are also performed out to complete the fabrication process, such as loophole interconnection and 3D integration^252^. Device illumination is also a factor that should be considered for FPA integration. The back-illuminated device is more conducive to the bonding of the array and ROIC. The opaque multiplexer will not reduce the effective light area of the device in the back-illuminated AlGaN-based structure, as shown in Fig. 16. In the case of ROIC, preamplifier techniques promote the development of high-performance FPAs. Due to a large dark current caused by background noise and the nonuniformity of the material, ROIC will face the problems of a small dynamic range and large spatial noise. Therefore, background and dark current suppression (BDS) circuits are required to reduce spatial noise and provide fast frame rates^253^. Various circuits are employed for FPAs, such as direct injection (DI) circuit^254–258^, capacitive transimpedance amplification input circuit (CTIA)^259–262^, and source follower per detector (SFD)^263–265^. DI yields poor performance at low flux, while CTIA yields high gains and is more complex. The SFD is the most common circuit in the infrared radiation astronomy field. In recent decades, the development of UV detectors and integrated circuits has jointly promoted the advancement of FPA imaging technology.

### Development of AlGaN-based solar-blind UV FPAs

In the back-illuminated Al_x_Ga_1-x_N-based solar-blind UV FPA, the imaging element is commonly composed of a detector array and a silicon-based CMOS ROIC (Fig. 16). The Al_x_Ga_1-x_N solar-blind UV array detects UV signals and converts the UV light emitted or reflected by the target into electrical signals. The silicon CMOS ROIC stores the electrical signals collected by the UV detector array in the integrating capacitor, and reads out the electrical signals distributed in space in a certain time sequence relationship to automatically complete two-dimensional (2D) imaging. When the Al_x_Ga_1-x_N-based UV FPA works in the solar-blind region of the solar spectrum under the atmosphere, there is no need or less use of UV filters, which can increase the UV light transmittance and improve the detectivity, due to its intrinsic solar-blind characteristic.

**Fig. 17:**
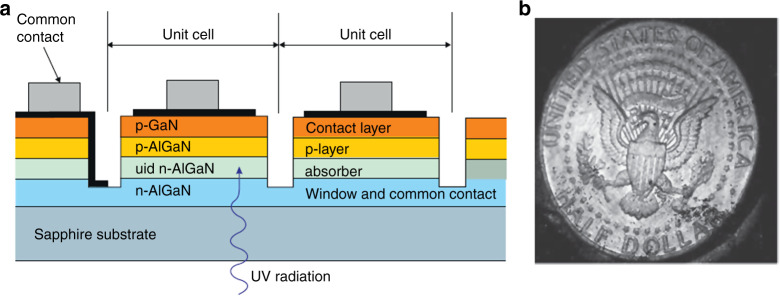
256 × 256 AlGaN UV FPA structure and imaging. **a** Schematic cross-sectional diagram of AlGaN-based FPA structure. **b** UV reflection image of a US dollar coin with the fabricated FPA. Reprinted with permission from Lamarre et al.^266^. Copyright WILEY-VCH Verlag Berlin GmbH

In 2001, Lamarre et al. reported back-illuminated 256 × 256 AlGaN UV FPAs with 30 × 30 μm^2^ unit pixels^266^. Figure 17a shows the schematic structure of the FPA. An Mg-doped p-type GaN cap layer is used for better ohmic contact. Figure 17b shows the UV reflection image of a coin by this FPA.

**Fig. 18:**
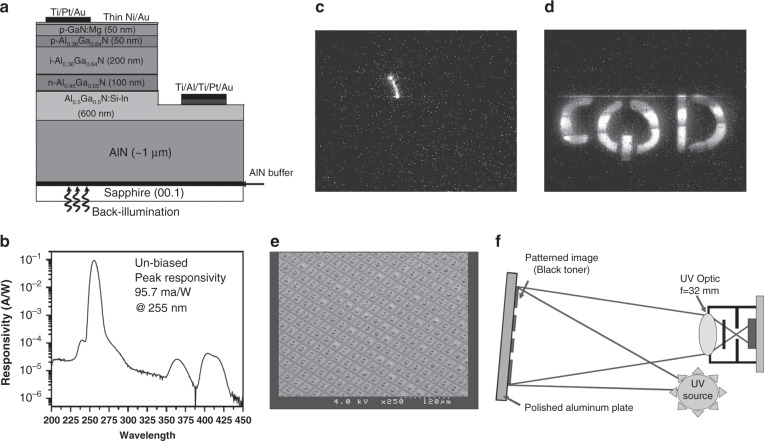
320 × 256 AlGaN UV FPAs and imaging technology. **a** Schematic structure of the AlGaN photodetector. **b** Spectral response at 0 V. **c** Electrical arc discharge image from FPA camera. **d** Paper-cutout image from FPA camera. **e** SEM image of FPA with indium bump. **f** Schematic diagram of Imaging geometry. Reprinted with permission from McClintock et al.^267^. Copyright 2005 SPIE

The Razeghi research group at Northwestern University in the United States also reported a 320 × 256 Al_x_Ga_1-x_N-based UV detector array in 2005^267^. The prepared FPA is composed of 320 × 256 arrays of 25 μm × 25 μm pixels with a period of 30 μm, as shown in Fig. 18e. There is a common n-type contact ring on the periphery of the array. Before combining FPA and ROIC, the electrical characteristics of individual pixels in the array were studied. The turn-on voltage of the pixel is 4.7 V, the series resistance is 4.3 kΩ, and the ideal factor of the device is 3.6. Figure 18a shows the schematic cross-sectional structure of the p-i-n AlGaN PD. The device exhibits a significant response peak at 255 nm under zero bias (Fig. 18b).

Due to the absorption of solar-blind light by ozone spheres, 280-nm light radiation is rare in nature. The imaging of the camera requires artificial scenes to cooperate. A simple scene can use shortwave UV light and a shadow mask props, these props form a certain shape and are then imaged by the camera. Figure 18d shows the image of the prop letter CQD. UV cameras can also image arcs and coronas, which can be used for the diagnosis of high-voltage equipment, and can also be used for military missile warnings, accurate positioning imaging of ships and port berthing in fog, and many other solar-blind imaging fields. Figure18c is a solar-blind image of a small flyback transformer with high-frequency arcs. The imaging geometry is illustrated in Fig. 18f. By improving the FPA processing methods (background difference), the pixel yield can be improved, thereby eliminating the existence of poor scan points.

For FPAs, important indicators for judging performance include pixel uniformity and anti-noise ability. Additionally, the parasitic illumination of multiplexers and cooled readout circuits also restrict the development of FPA imaging technology. Reine et al. reported a back-illuminated 256 × 256 hybrid AlGaN-based FPAs for solar-blind UV detection^268^. The detection wavelength covers the range from 260 to 280 nm. The response nonuniformities of the FPA are as low as 2.5%. They obtained the best pixel with a low noise equivalent irradiance of 90 photos/pixel at 1 Hz. Reverchon et al.^269^ demonstrated an FPA of 320 × 256 Al_0.45_Ga_0.55_N Schottky photodiode pixels. The multiplex process with ROIC was implemented using a black matrix at room temperature. Using a basic black glass to reduce noise, a UV/visible rejection ratio of four orders of magnitude was achieved.

**Fig. 19:**
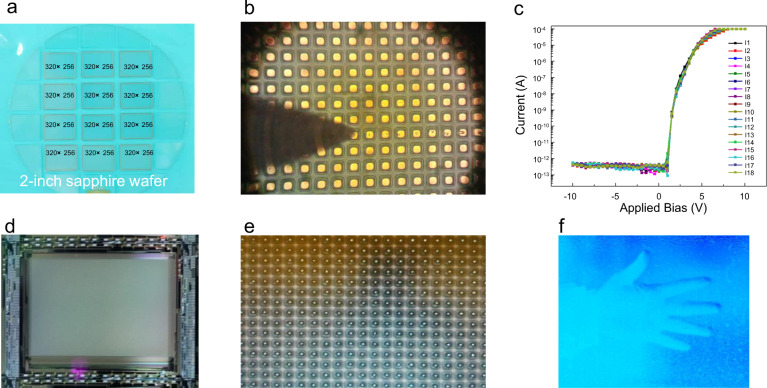
AlGaN p-i-n solar-blind UV FPAs and imaging by Nanjing University. **a** 320 × 256 AlGaN FPAs on 2-inch sapphire wafer. **b** Optical images of AlGaN focal plane arrays. **c** I-V characteristics of individual photodetector. **d** Silicon driver IC. **e** Optical images of array with Indium bumps. **f** Solar-blind ultraviolet images taken from the AlGaN FPA camera

Hai Lu et al. of Nanjing University also fabricated AlGaN p-i-n FPAs with 320 × 256 pixels. As shown in Fig. 19a, b, FPAs are prepared on a 2-inch sapphire substrate with a pixel size of 25 × 25 μm^2^. The I-V characteristics show superior pixel performance and consistency, as shown in Fig. 19c. The arrays are integrated with silicon driver circuits by indium bumps (Fig. 19d, e). This AlGaN FPA camera can describe solar-blind UV imaging, and a hand-shaped picture is exhibited in Fig. 19f.

In terms of substrate material, the AlGaN detector that is prepared on sapphire can be used to fabricate FPA, and AlGaN devices on Si can also be manufactured for FPA imaging. Malinowski et al. reported the fabrication of an AlGaN-based FPA of 256 × 256 pixels integrated with a CMOS readout chip^270^. A thin silicon layer was left to block light with wavelengths higher than 20 nm. The proposed FPA was sensitive down to 1-nm wavelength, thus achieving extreme UV light imaging. Cicek et al.^118^ proposed a scheme of laterally epitaxial AlGaN/AlN on silicon and fabricated an AlGaN hybrid FPA with an indium bump. Unlike sapphire epitaxy, it is necessary to remove the Si substrate with acid after attachment to prevent Si from absorbing solar-blind UV light. The p-i-n unit PD pixel yielded a low dark current density of 1.6 × 10^−8^ A/cm^2^ at 10 V, which confirmed the feasibility of AlGaN growth on silicon.

**Fig. 20:**
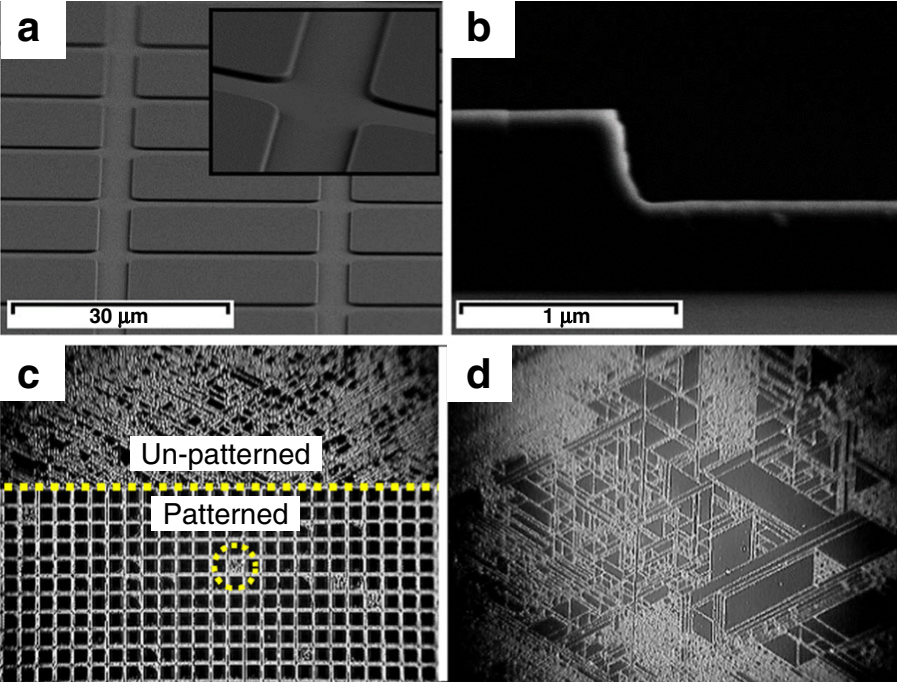
Reduced area epitaxy for the growth of AlGaN photodetector pixels. **a** SEM image of patterned AlN, mesa size: 26 μm × 26 μm, periodicity: 30 μm. **b** Cross-sectional SEM image of sidewall. Implemented AlGaN UV photodetector grown on patterned (**c**) and unpatterned (**d**) AlN template. Reprinted with permission from Cicek et al.^271^. Copyright 2013 American Institute of Physics

Because the FPA is composed of individual pixel units, the reduced area epitaxy (RAE) method can be used for the growth of the PD structure. As shown in Fig. 20a, patterned AlN fabricated after dry etching was used to grow AlGaN structure. An etching depth of 300 nm is shown in the cross-sectional image in Fig. 20b. The biaxial strain can be released by the patterned squares to improve the crystal quality. A comparison of the RAE and non-RAE epitaxial materials shows that the patterned epitaxial pixels are nearly crack-free, while the counterpart exhibits many cracks, as shown in Fig. 20c, d. Cicek et al. obtained 97% crack-free pixels through the RAE method^271^.

Due to the improvement of AlGaN material preparation technology and device performance, AlGaN has become a superior alternative material for the development of small-size, lightweight, and low-power solar-blind FPAs. To express the development process of AlGaN-based FPAs more clearly, we reviewed and summarized the results of reported AlGaN-based FPAs in recent years, as shown in Table 4.

**Table 4:** Summary of reported AlGaN-based focal plane arrays

Material (Active region)	Pixel structure	Pixel points	Operating wavelength (nm)	Year	Reference
AlGaN	p-i-n	256 × 256	265–285	2001	Lamarre et al.^266^
Al_0.32_Ga_0.68_N	p-i-n	320 × 256	280	2005	McClintock et al.^267^
Al_0.36_Ga_0.64_N	p-i-n	320 × 256	278	2005	McClintock et al.^285^
Al_0.45_Ga_0.55_N	p-i-n	256 × 256	260–280	2006	Reine et al.^268^
Al_0.08_Ga_0.92_N	p-i-n	8 × 8	330–350	2006	Kim et al.^286,287^
Al_0.45_Ga_0.55_N	Schottky	320 × 256	280	2007	Reverchon et al.^269^
Al_0.59_Ga_0.41_N	p-i-n	128 × 128	233–258	2008	Yuan et al.^62^
Al_0.43_Ga_0.57_N	Schottky	320 × 256	260–290	2009	Duboz et al.^288^
Al_0.4_Ga_0.6_N with Si-layer	Schottky	256 × 256	1–33	2011	Malinowski et al.^270^
Al_0.45_Ga_0.55_N	p-i-n	320 × 256	290	2013	Cicek et al.^118^
Al_0.4_Ga_0.6_N	p-i-n	320 × 256	275	2013	Cicek et al.^271^
Al_0.4_Ga_0.6_N	p-i-n	320 × 256	278	2015	McClintock et al.^288^
AlGaN	p-i-n	320 × 256	200–400	2018	Lakovleva et al.^289^
Al_0.43_Ga_0.57_N	p-i-n	640 × 512	280	2020	Rehm et al.^290^

It can be concluded that most AlGaN detectors in FPAs exhibit a p-i-n structure. The vertical structure can be used to fabricate the back-illuminated PD, which is beneficial to the flip-on-chip process. Currently, solar-blind UV imaging of 320 × 256 pixels can typically be achieved. However, FPAs composed of avalanche optoelectronic devices are rarely reported, which can be attributed to the stricter requirements of higher device uniformity and yield in AlGaN APDs. Large-area UV imaging arrays are also not as sharp as visible and infrared light. With the sustained improvement of the epitaxial technique and device performance, the technology of AlGaN solar-blind UV imaging will likely develop quickly. All permissions and copyrights of reprint figures in this review are provided in the supplementary material.

## Summary and prospects

Although considerable progress has been made with AlGaN-based solar-blind UV PDs over the past two decades, the performances of these devices have yet to meet expectations, particularly with single photon detection. The biggest bottleneck in the development of AlGaN-based solar-blind UV PDs is the high-quality film epitaxy of high-Al-content AlGaN alloys. Many ingenious and thoughtful epitaxy methods or techniques mentioned above such as high-low AlN buffers, SL dislocation filter, or stress control layer, PALE method, ELO techniques, have substantively improved the crystal quality of AlGaN alloys. Additionally, bulk AlN single-crystal substrates can mitigate serious lattice mismatches, and thermal mismatches can occur when using sapphire substrates to fabricate AlGaN films, but large-area AlN bulk single crystal is not commercially available. Using an AlN or AlGaN nanopatterned template based on a sapphire substrate is a promising way to fabricate high-quality AlGaN films combined with the ELO technique.

However, the threading dislocation density in AlGaN materials is typically above 1 × 10^8^ cm^−2^ at present, which is six orders of magnitude higher than that of SiC, a relatively mature material in developing UV SPAD. Therefore, it is very difficult to prepare materials that meet the requirements of SPAD only by relying on the existing growth methods and equipment. To obtain further high-quality AlGaN materials, certain challenges must be overcome, such as improving the growth chamber and optimizing the growth conditions from the perspective of growth kinetics. The fabrication of p-AlGaN suffers from low Mg acceptor doping efficiency in addition to serious crystal quality problem. Although many methods as aforementioned have been developed to suppress the self-compensation process, increase the solubility of Mg atom, and reduce the activation energy of Mg acceptor in AlGaN, how to guarantee achieving a high HC and a high crystal quality of p-AlGaN simultaneously requires further research and better methods.

Certainly, structural design and fabrication processes are also important factors in developing low-noise and high-gain AlGaN-based solar-blind UV PDs, particularly in this heterostructure system with a strong polarization effect and large lattice mismatch. Polarization field control and energy-band cutting technologies will become increasingly important means for the design of AlGaN heterostructure PDs. Bevel mesa and field plate terminal technologies can effectively uniform the electric field distribution and are helpful to reduce the dark current and prevent early breakdown of a device.

Moreover, further research on device physics is necessary to achieve state-of-the-art AlGaN-based solar-blind UV PDs. Under the critical breakdown electric field, the contribution of different types of dislocations to the tunneling leakage current remains unclear, particularly in a high-dislocation-density material system. The effect of dislocations on impact ionization has also rarely been studied. Understanding these physics mechanisms will help us to take more effective methods to solve material fabrication and process questions. The experimental values of the carrier impact ionization coefficient in AlGaN materials have not yet been extracted, which affects the accurate design and simulation of the device structure.

Regarding application field, AlGaN FPAs play an irreplaceable role in solar-blind UV imaging. Despite marked progress in AlGaN FPAs, the uniformity and scale of arrays must be further improved. In particular, avalanche FPAs for detecting weak signals is still confronted with huge challenges. With the development of epitaxial and PD technology, the fabrication of large-scale AlGaN solar-blind UV FPAs is anticipated in the near future.

## Supplementary information

Permission and copyright
