# Author Correction: Progress on AlGaN-based solar-blind ultraviolet photodetectors and focal plane arrays

**DOI:** 10.1038/s41377-021-00690-8

**Published:** 2021-12-07

**Authors:** Qing Cai, Haifan You, Hui Guo, Jin Wang, Bin Liu, Zili Xie, Dunjun Chen, Hai Lu, Youdou Zheng, Rong Zhang

**Affiliations:** 1 Key Laboratory of Advanced Photonic and Electronic Materials, School of Electronic Science and Engineering, Nanjing University, Nanjing, 210093 China; 2 Collaborative Innovation Center for Optoelectronic Semiconductors and Efficient Devices, Department of Physics, Xiamen University, Xiamen, 361005 China; 3 Institute of Future Display Technology, Tan Kah Kee Innovation Laboratory, Xiamen, 361102 China

**Keywords:** Optical materials and structures, Other photonics

Correction to: *Light: Science & Applications*

10.1038/s41377-021-00527-4 published online 30 April 2021

Following publication of the article^1^, it is reported that this article contains some errors. The correction details are listed below.

(1) Figure 19 has been updated as below.

**Fig. 19:**
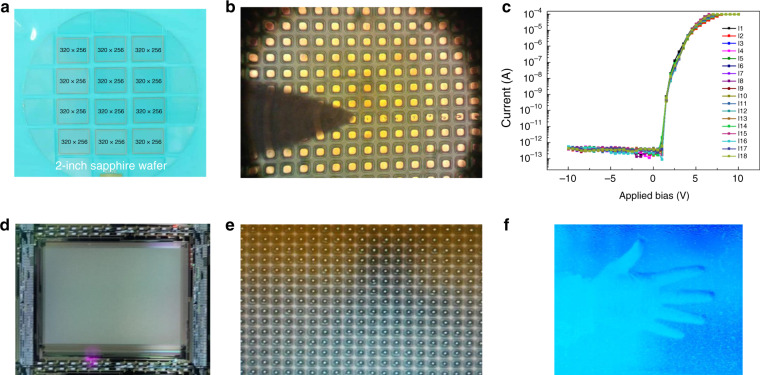
AlGaN p-i-n solar-blind UV FPAs and imaging by Nanjing University. **a** 320 × 256 AlGaN FPAs on 2-inch sapphire wafer. **b** Optical images of AlGaN focal plane arrays. **c** I-V characteristics of the individual photodetector. **d** Silicon driver IC. **e** Optical images of the array with Indium bumps. **f** Solar-blind ultraviolet images taken from the AlGaN FPA camera
